# Structural insights into phosphatidylethanolamine formation in bacterial membrane biogenesis

**DOI:** 10.1038/s41598-021-85195-5

**Published:** 2021-03-11

**Authors:** Gyuhyeok Cho, Eunju Lee, Jungwook Kim

**Affiliations:** Department of Chemistry, Gwangju Institute of Science and Technology, Gwangju, 61005 Republic of Korea

**Keywords:** Biochemistry, X-ray crystallography

## Abstract

Phosphatidylethanolamine (PE), a major component of the cellular membrane across all domains of life, is synthesized exclusively by membrane-anchored phosphatidylserine decarboxylase (PSD) in most bacteria. The enzyme undergoes auto-cleavage for activation and utilizes the pyruvoyl moiety to form a Schiff base intermediate with PS to facilitate decarboxylation. However, the structural basis for self-maturation, PS binding, and decarboxylation processes directed by PSD remain unclear. Here, we present X-ray crystal structures of PSD from *Escherichia coli*, representing an apo form and a PE-bound complex, in which the phospholipid is chemically conjugated to the essential pyruvoyl residue, mimicking the Schiff base intermediate. The high-resolution structures of PE-complexed PSD clearly illustrate extensive hydrophobic interactions with the fatty acyl chains of the phospholipid, providing insights into the broad specificity of the enzyme over a wide range of cellular PS. Furthermore, these structures strongly advocate the unique topology of the enzyme in a lipid bilayer environment, where the enzyme associates with cell membranes in a monotopic fashion via the N-terminal domain composed of three amphipathic helices. Lastly, mutagenesis analyses reveal that *E. coli* PSD primarily employs D90/D142–H144–S254 to achieve auto-cleavage for the proenzyme maturation, where D90 and D142 act in complementary to each other.

## Introduction

Phosphatidylethanolamine is a major lipid component of cellular membranes in a wide range of organisms^1,2^. In *Escherichia coli*, PE accounts for 70–80% of total membrane lipids, and it is exclusively synthesized by phosphatidylserine decarboxylase (PSD) on the cytoplasmic side of the inner membrane^3–6^. PSD decarboxylates the substrates on the side of its bound leaflet^7,8^, generating an asymmetric distribution of PE across the inner membrane. A difference has been reported in the chemical and structural properties of PS and PE within the membrane; the molecular structure of PS exhibits a cylindrical shape with a net negatively charged head group, while PE assumes an inverted cone shape with a net neutral head group^9,10^. Therefore, the concentration gradient of PE across the membrane is crucial for proper folding, topological flipping, and the function of membrane proteins such as lactose permease LacY^11^. Notably, PSD is essential for numerous pathogenic bacteria, including *E. coli, Vibrio cholerae, Pseudomonas aeruginosa, Acinetobacter baumannii,* and *Helicobacter pylori*^12^.

In humans, PE is the second most abundant phospholipid in the mitochondrial membrane (30–35%), after phosphatidylcholine (40–46%)^13^, which is mainly synthesized by PISD (equivalent to bacterial PSD) located on the inner membrane (IM) of the mitochondria^2,14^. Meanwhile, the majority of non-mitochondrial PE is synthesized via the cytidine diphosphate (CDP)-ethanolamine pathway in the ER. In mammalian cells, the CDP-ethanolamine-dependent pathway has been shown to favor phospholipids containing mono- or di-unsaturated fatty acyl chains connected to the *sn-2* position of the glycerol backbone, whereas PISD preferentially decarboxylates PS with a polyunsaturated acyl chain at the *sn-2* position or with hydrophilic diacyl chains^15,16^. In *Saccharomyces cerevisiae*, most cellular and mitochondrial PE is provided by PSD1p, which is located in the mitochondrial inner membrane^17^, whereas PE in the Golgi/vacuolar compartment is synthesized by PSD2p. PE produced by PSD1p is essential for the function of the cytochrome bc1 complex^18^. These pathways jointly provide various molecular pools of PE to different cellular compartments, with each biosynthetic pathway uniquely contributing to membrane biogenesis.

**Figure 1:**
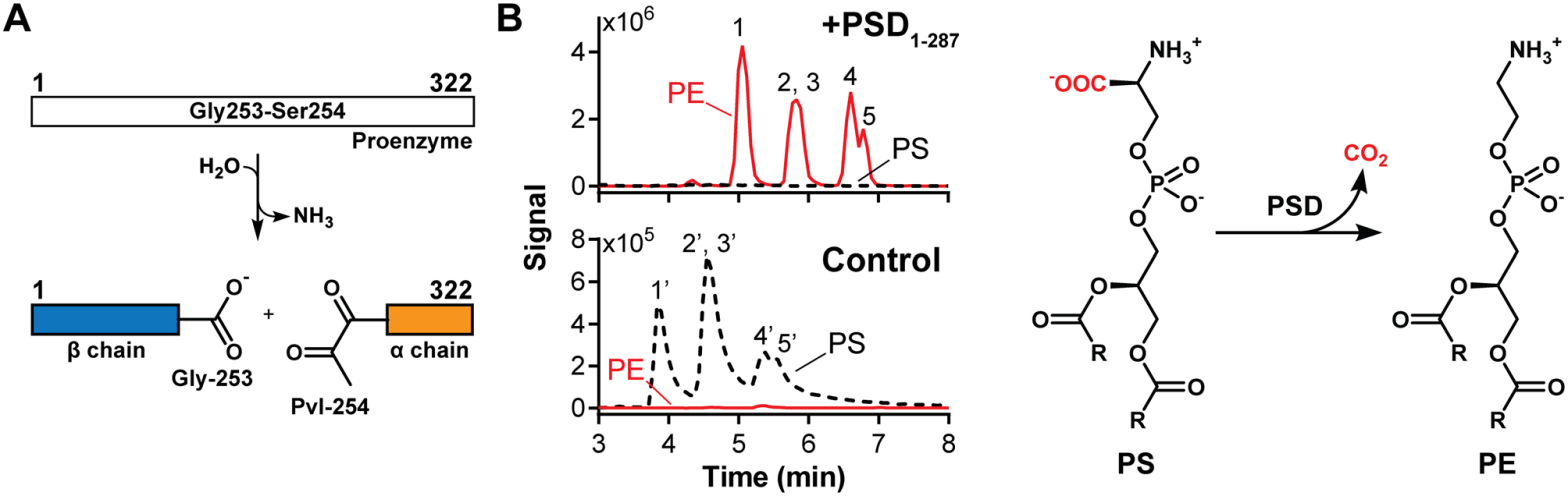
Auto-cleavage and PS decarboxylation activity of *E. coli* PSD. (**A**) A schematic of auto-cleavage. Pvl, pyruvoyl residue. (**B**) LC–MS analysis of PS decarboxylation by PSD_1–287_. Numbers in the chromatogram correspond to individual PE or PS molecular species: 1–5 represent 36:4, 34:2, 36:3, 34:1, and 36:2 PE (length of acyl-chain:degree of saturation), respectively; 1′-5′ denote for equivalent acyl-chain PS. Scheme of PS decarboxylation is shown on the right.

PSD is translated as an inactive proenzyme and undergoes self-cleavage between a conserved Gly-Ser pair to become an active αβ-heterodimer (Fig. 1A)^19^. This maturation step results in the post-translational modification of the N-terminus serine to pyruvoyl in the α-subunit, which is an indispensable prosthetic group for decarboxylation activity^20^. PSD is a serine protease that employs a classic catalytic triad composed of Asp-His-Ser, where nucleophilic serine attacks the scissile peptide bond to produce α- and β-chains^21^. Through mutagenesis experiments, D139-H198-S308 and D210-H345-S463 were identified as the catalytic triads for PSD in *Plasmodium knowlesi*^22^ and *S. cerevisiae*^23^, respectively. After the proenzyme undergoes auto-proteolysis, an active complex is formed between the α- and β-chains, which are mainly responsible for catalytic activity and membrane association, respectively. The reaction mechanism for PSD was proposed based on that of pyruvoyl-dependent histidine decarboxylase^24^, where the amine group of PS and the α-carbonyl carbon of pyruvoyl form a Schiff base intermediate, followed by decarboxylation^24^.

The first crystal structures of PSD from *E. coli* in apo- and lipid-bound forms have been reported recently, which offer valuable information regarding the membrane-associated mechanism, substrate binding, and determinants critical for catalytic activity^25^. The lipid-bound structure displays covalent conjugation of PE to the pyruvoyl residue; however, a rather low resolution (3.60 Å) limits the identification of acyl chain moieties of the bound lipid molecule. Here, we report two crystal structures of *E. coli* PSD, representing apo states at resolutions of 1.90 and 2.63 Å, along with two PE-bound structures of PSD at resolutions of 2.12 and 2.70 Å, which mimic the Schiff base intermediate formed between the pyruvoyl group and a phospholipid. Our high-resolution structures allow the identification of the exact locations where intermolecular interactions occur between the enzyme and the bound phospholipid molecule via diacyl chains in particular. Structure-guided mutagenesis analyses confirmed the key residues involved in phospholipid recognition, decarboxylation of PS, and maturation of PSD. In particular, we identified the crucial residues required for the activation of proenzymes, which have not been characterized for *E. coli* PSD.

## Results

To facilitate crystallization and structure determination, we employed a recombinant *E. coli* PSD with the last 35 residues removed (PSD_1–287_). The amino acid sequences of this region are missing in most bacteria, except for certain species of *Enterobacterales* (Supplementary Fig. S1). The in vitro activity of PSD_1–287_ was tested using soy PS as a substrate, and five major phospholipid species were quantitatively analyzed via LC–MS (Fig. 1B and Supplementary Fig. S2A). Under our assay conditions, it is estimated that over 95% of PS is enzymatically converted to PE. This result is comparable to that of the full-length wild-type enzyme (Supplementary Fig. S2B), indicating that truncation of the C-terminal residues does not significantly contribute to decarboxylation activity.

### Overall feature of apo structures

**Figure 2:**
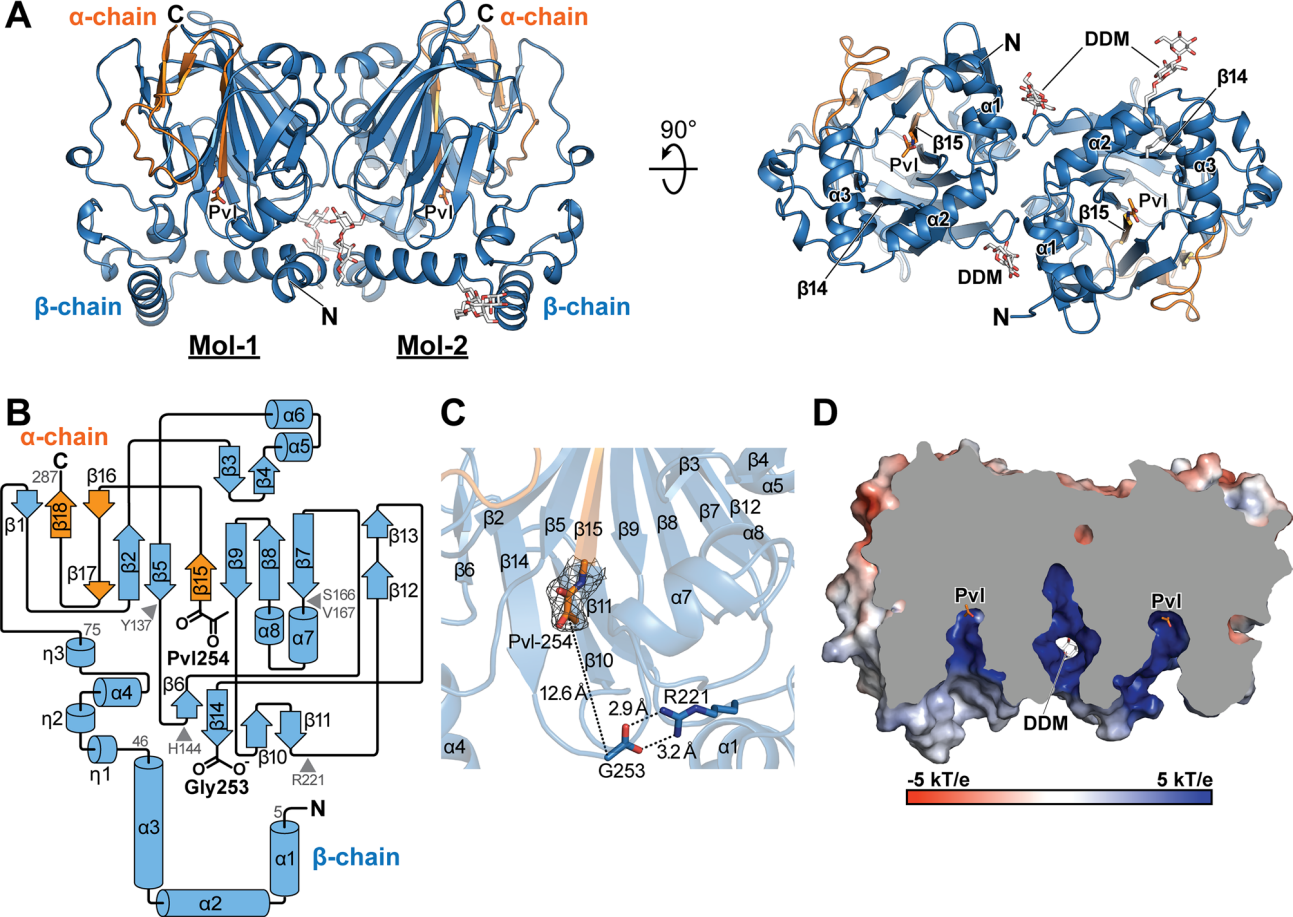
Overall crystal structure of apo *E. coli* PSD. (**A**) A structure of apo α_2_β_2_ heterotetramer. Left and right, two views are related to 90° rotations. The beta chain is illustrated in sky blue, alpha chain in orange, and DDM as stick in white. Pvl, pyruvoyl residue; DDM, dodecyl-beta-d-maltoside. (**B**) Topology of the αβ-heterodimer. An identical color scheme is used as in (**A**). (**C**) A close-up view of the auto-cleaved site. A *Fo-Fc* omit map for pyruvoyl residue contoured at 3 σ is displayed in mesh. Other residues and DDM were omitted for clarity. Distances between C_α_ of Pvl-254 and Gly-253 and two interactions in salt bridge designated as dashed lines, which were calculated by averaging values from 6 Apo-PSD protomers. (**D**). Sliced view of Apo-PSD1 is shown to highlight the funnel-shaped active site. Electrostatic potential is mapped on the surface of the enzyme. Two pyruvoyl residues on the active sites and DDM molecules are shown in sticks.

Using native and selenomethionine-derivatized recombinant PSD_1–287_, the crystal structures of apo PSD_1–287_ were determined from two crystal forms at resolutions of 1.90 and 2.63 Å, which are described as Apo-PSD1 and Apo-PSD2, respectively. The crystallographic statistics are summarized in Table 1. The asymmetric unit of Apo-PSD1 contains two αβ-heterodimers, whereas that of Apo-PSD2 contains four (Supplementary Fig. S3). The biological assembly of PSD_1–287_ appears to be a dimer of αβ-heterodimers, (αβ)_2_, which is commonly identified in all four structures presented herein (Fig. 2A). The heterotetrameric form of PSD_1–287_ in solution was consistent with the results of the SEC-MALS analysis (Supplementary Fig. S4). Two out of three molecules of *N*-dodecyl-b-maltoside (DDM), a detergent used to solubilize the enzyme, were located at the interface between two adjacent β-subunits, augmenting the dimerization of αβ-heterodimers in Apo-PSD1. No detergent molecules were modeled in Apo-PSD2 structure. The α and β subunits form a tight 1:1 complex in each heterodimer with an average interface area of 1790 Å^2^, where 29 out of 34 residues from the α subunit participate in forming the dimerization interface. Using the N-terminal sheet composed of residues from Pvl-254 to Ala-261, the α-subunit was integrated between two anti-parallel sheets of the β-subunit (Fig. 2B). The N-terminal pyruvoyl residue (Pvl-254) of the α-subunit was unambiguously identified in both apo structures (Fig. 2C). Gly-253 of the β subunit, which is no longer connected to Pvl-254 after auto-cleavage, is approximately 12.6 Å apart when the distance between two C_α_s is measured, and it forms a salt bridge with the side chain of Arg-221. Consequently, Pvl-254, which is located at the base of the funnel-shaped active site, is open for substrate binding (Fig. 2D).

**Table 1:** Crystallographic data collection and refinement statistics.

	Apo-PSD1 (PDB: 7CNW)	Apo-PSD2 (PDB: 7CNX)	8PE-PSD (PDB: 7CNY)	10PE-PSD (PDB: 7CNZ)	Apo-PSD1 (SeMet)
**Data collection**					
Wavelength (Å)	0.9801	0.9793	0.9796	0.9796	0.9793
Space group	P212121	P212121	P212121	P212121	P212121
**Cell dimensions**					
*a*, *b*, *c* (Å)	77.44, 79.82, 147.09	79.90, 101.85, 170.10	77.15, 79.46, 146.77	79.72, 102.37, 168.99	77.35, 78.73, 147.46
α, β, γ (°)	90.00, 90.00, 90.00	90.00, 90.00, 90.00	90.00, 90.00, 90.00	90.00, 90.00, 90.00	90.00, 90.00, 90.00
Resolution (Å)	49.03–1.90 (1.94–1.90)	48.78–2.63 (2.73–2.63)	29.35–2.12 (2.18–2.12)	29.47–2.70 (2.82–2.70)	34.73–2.12 (2.18–2.12)
*R*_sym_	0.132 (2.802)	0.146 (2.609)	0.122 (2.231)	0.185 (2.649)	0.267 (3.540)
*R*_pim_	0.037 (0.845)	0.055 (0.964)	0.050 (0.896)	0.052 (0.757)	0.072 (0.939)
CC_1/2_	0.999 (0.369)	0.998 (0.441)	0.999 (0.504)	0.999 (0.531)	0.997 (0.389)
$\left\langle {I/\sigma I} \right\rangle$	14.6 (1.0)	12.3 (1.0)	10.9 (1.0)	12.6 (1.0)	10.1 (1.0)
**No. of reflections**					
Observed	1,009,308	336,646	351,626	529,506	750,538
Unique	71,625 (3952)	42,052 (4328)	51,984 (4215)	38,760 (4666)	51,324 (4116)
Completeness (%)	98.8 (86.8)	100.0 (100.0)	99.9 (99.9)	99.9 (100.0)	99.1 (98.7)
Multiplicity	14.1 (11.4)	8.0 (8.2)	6.8 (7.0)	13.7 (13.1)	14.6 (14.9)
**Refinement**					
Resolution (Å)	41.81–1.90	48.83–2.63	28.65–2.12	29.43–2.70	
No. of reflections	67,965	39,931	49,340	36,717	
*R*_work_/*R*_free_	0.212/0.243	0.231/0.288	0.238/0.274	0.227/0.276	
No. of αβ-heterodimer in ASU	2	4	2	4	
Protein residues	571	1130	568	1131	
**No. of atoms**	4717	8453	4584	8680	
Protein	4450	8439	4434	8655	
Detergent/ion	88	–	60	15	
Water	179	14	90	10	
***B-factors (Å***^**2**^**)**	42.89	71.24	51.19	74.18	
Protein	42.45	71.28	51.14	74.17	
Detergent/ion	71.49	–	68.09	98.39	
Water	42.14	51.36	42.27	52.06	
**R.m.s deviations**					
Bond lengths (Å)	0.010	0.007	0.008	0.007	
Bond angles (°)	1.579	1.430	1.538	1.514	
**Ramachandran**					
Favored (%)	96.3	95.1	96.4	93.9	
Allowed (%)	3.7	4.5	3.6	5.8	
Outliers (%)	0	0.4	0	0.4	

Each dataset was collected from a single crystal. Values in parentheses are for highest-resolution shell.

### Identification of the membrane association domain

**Figure 3:**
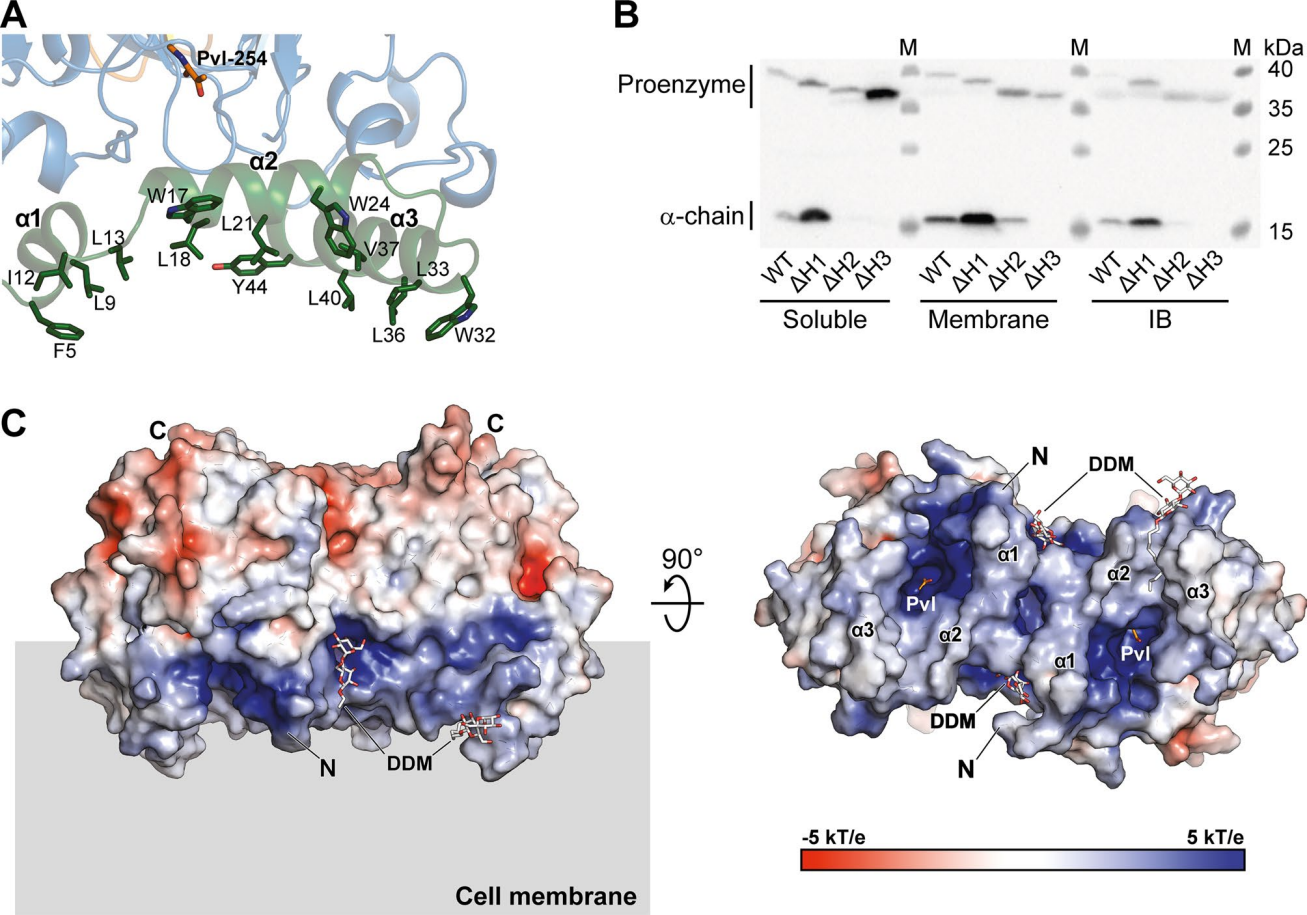
N-terminal three-helices are crucial for membrane binding and auto-cleavage. (**A**) Amphipathic nature of N-terminal helices are displayed in light green and the hydrophobic residues are labeled and highlighted in dark green. (**B**) Membrane association assay of PSD full-length (WT, 1–322), α1-helix (ΔH1, 13–322), α1–2 (ΔH2, 30–322), and α1–3 truncated (ΔH3, 46–322). The proteins were heterologously expressed in *E. coli*. The cells were lysed by sonication in non-detergent solution (PBS, phosphate-buffered saline), and separated into soluble, membrane, and inclusion body (IB) fractions by sequential rounds of centrifuge and ultracentrifuge. Each fractions were analyzed by 12% SDS-PAGE and western blotting using anti-histag antibody. *Lane M* ladder marker. (**C**) Distribution of electrostatic potential on αβ-heterotetramer. Left and right, two views are same as in Fig. 2A. The surface with positive charge is displayed in blue, negative in red, and neutral in white. Approximate location of cell membrane is shown as gray box. N-terminal three-helices are designated on the left panel.

The three N-terminal helices contributed by each β-subunit lie in a plane, where hydrophobic residues are aligned on one side of the helices (Fig. 3A). Therefore, six N-terminal helices of the heterotetramer are ideally shaped to make contact with the coplanar lipid layers of the inner membrane of *E. coli*. To investigate the role of the three helices in membrane association, the cellular location of the wild-type protein and a series of truncated PSD in the N-terminal domain were examined via immunoblotting of soluble, membrane, and insoluble fractions after cell lysis (Fig. 3B). The mutant ΔH1 lacks only the N-terminal helix 1, whereas ΔH2 lacks helices 1 and 2, and ΔH3 is devoid of helices 1–3. Most wild-type proteins were detected in the membrane fraction, whereas ΔH1 exhibited enrichment in the soluble fraction, as well as in the membrane fraction. Additional truncations led to incomplete auto-cleavage of the proenzyme in ΔH2 and ΔH3, and the level of mature PSD was low overall. Notably, uncleaved ΔH3 was enriched in the soluble fraction, and the cleaved form was not detected at a significant level in all fractions. The concentration of hydrophobic residues on the surface of the N-terminal helices further supports that the N-terminal domain is a *hot spot* for membrane association (Fig. 3C and Supplementary Fig. S5). Interestingly, DDM molecules identified in the structure of Apo-PSD1 interact with the positively charged surface of PSD through the relatively polar disaccharide moiety, whereas the dodecyl tail of the detergent interacts with the hydrophobic surface of the enzyme encompassing the N-terminal helices (Supplementary Fig. S6). These molecular interactions may mimic those occurring on the cellular membranes, where PSD is partially embedded within a single layer of phospholipids using the N-terminal helical domain.

### Phospholipid-bound structures

**Figure 4:**
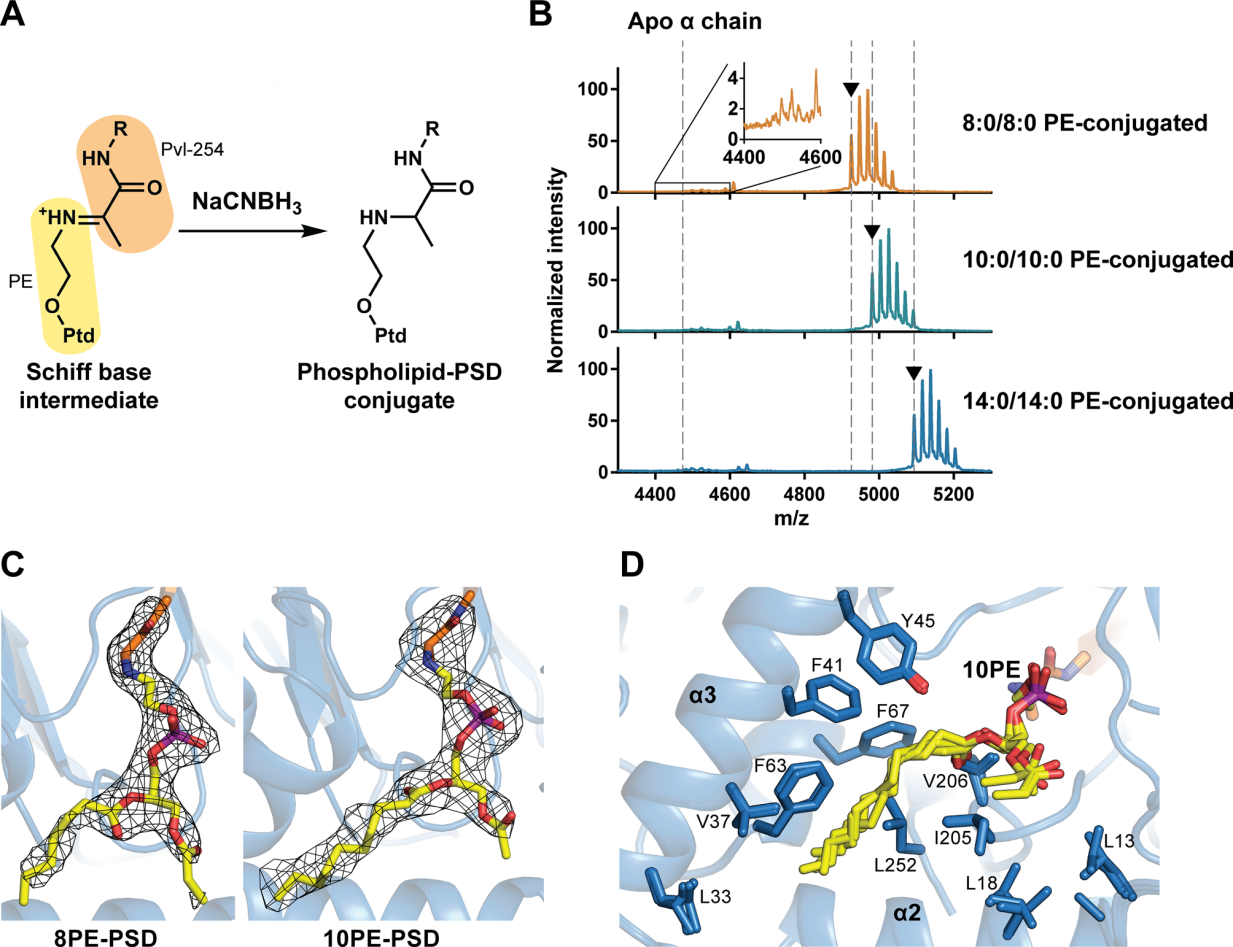
Phospholipid-bound structures. (**A**) A scheme of NaCNBH_3_-dependent reduction of Schiff base. Ptd, phosphatidyl moiety (**B**) MALDI-ToF analysis of phospholipid conjugation on α-chain. The MS peaks of apo and modified α-chains are marked as gray dashed lines and black arrows for comparison of the *m/z* values. Observed and calculated *m/z* values of conjugates are 4925.6, 4926.6 (8:0/8:0 PE-conjugated), 4981.8, 4980.7 (10:0/10:0 PE-conjugated), and 5093.8, 5092.8 (14:0/14:0 PE-conjugated), respectively. (**C**) A zoomed-in view of linked phospholipid from 8PE-PSD (Left) and 10PE-PSD (Right). A *Fo–Fc* omit map of the phospholipid is illustrated as mesh contoured at 2.5 σ. Carbon atoms of the lipid are displayed in yellow, oxygen in red, nitrogen in blue, and phosphorous in purple. (**D**) Overlay of the acyl-chains of bound lipid in the hydrophobic pocket from all four subunits of 10PE-PSD structure. Hydrophobic residues surrounding the acyl-chains are labeled and highlighted as light blue stick.

To identify the molecular determinants required for substrate recognition and binding, we first attempted to form a stable covalent linkage between PSD and a phospholipid via sodium cyanoborohydride (NaCNBH_3_)-dependent reduction of a Schiff base intermediate (Fig. 4A)^19^. Conjugation was tested using 8:0/8:0 PE (8PE), 10:0/10:0 PS (10PS), and 14:0/14:0 PS (14PS) with full-length and truncated PSD, and the results were analyzed using matrix-assisted laser desorption/ionization time-of-flight (MALDI-TOF) mass spectrometry. Incubation of PSD with a phospholipid increased the mass-to-charge ratio (*m*/*z*) of the α-subunit by 450.5, 506.7, and 618.8 for 8PE, 10PS, and 14PS, respectively, which is consistent with the formation of conjugates with the corresponding PE (Fig. 4B). The results indicate that the reduction of the Schiff base occurred after decarboxylation. MS data confirmed the successful modification of the protein with an efficiency greater than 94%: 98%, 97%, and 94% conjugation for 8PE, 10PE, and 14PE, respectively. Among these samples, 8PE- and 10PE-linked PSD_1–287_ yielded crystals, which diffracted to 2.12 Å and 2.70 Å resolution, respectively. These X-ray diffraction data were used to determine the structures shown in Fig. 4C; they are further denoted as 8PE-PSD and 10PE-PSD, respectively. The asymmetric unit of 8PE-PSD contains two αβ-heterodimers, whereas that of 10PE-PSD contains four.

The overall conformation of PSD in both PE-bound structures is highly homologous to that in apo structures (r.m.s.d. of C_α_s = 0.218 Å for 8PE-PSD; 0.399 Å and 0.390 Å for the 10PE-PSD αβ-heterotetramer). Similar to Apo-PSD1, one of the two DDM molecules was modeled at the interface between the two β-subunits in 8PE-PSD. Charge distribution on the enzyme surface demonstrates that the substrate-binding site creates a predominantly hydrophobic environment optimal for accommodating fatty acyl chains of glycerophospholipids (Fig. 4D). Solid electron densities were observed in all subunits of PE connected to Pvl-254. However, the quality of local electron densities around fatty acyl chains varies from one subunit to another. For example, in one αβ-heterodimer of 8PE-PSD, 12 out of 16 carbon atoms could be modeled in the fatty acyl group of 8PE, whereas only 9 were traceable in the other. Although we were able to observe the electron densities of two fatty acyl chains connected to the glycerol backbone, the *sn*-1 and *sn-*2 positions on the glycerol backbone could not be determined unambiguously with the present data even at a resolution of 2.12 Å. The assignment was based on the electron density in chain B of the 8PE-PSD structure, which was considered the best. The *sn*-2 acyl chains were modeled in close proximity to the hydrophobic protein surface, which was composed of Val-37, Phe-41, Phe-63, Phe-67, and Leu-252, with *sn*-1 acyl chains extending near the surface defined by Leu-13, Leu-18, and Thr-204. In general, the fatty acyl chain at the *sn*-2 position is more ordered than that connected to *sn*-1 in the present structure. For instance, the entire *sn*-2 acyl chains could be modeled in 10PE-PSD, whereas most *sn*-1 acyl chains were disordered because of the relatively shallow binding pocket. In both structures, the *sn*-2 acyl chains extend to the hydrophobic surface of the N-terminal helical domain, where the membrane association is presumed to occur, as described earlier. The *sn*-2 chain-binding site, which is composed of hydrophobic residues, allows for non-specific binding with a fatty acyl chain. This relaxed specificity is expected to be effective for the *sn*-1 chain binding site as well, in which the docked fatty acyl chain exhibits a greater degree of conformational flexibility. The binding mode of fatty acyl chains observed in our structures reflects the intrinsic nature of PSD-lipid interactions and are not crystallization artefacts, since there are no neighboring symmetrically related molecules, which would have affected the protein-lipid interactions.

### Recognition of phosphoserine moiety

**Figure 5:**
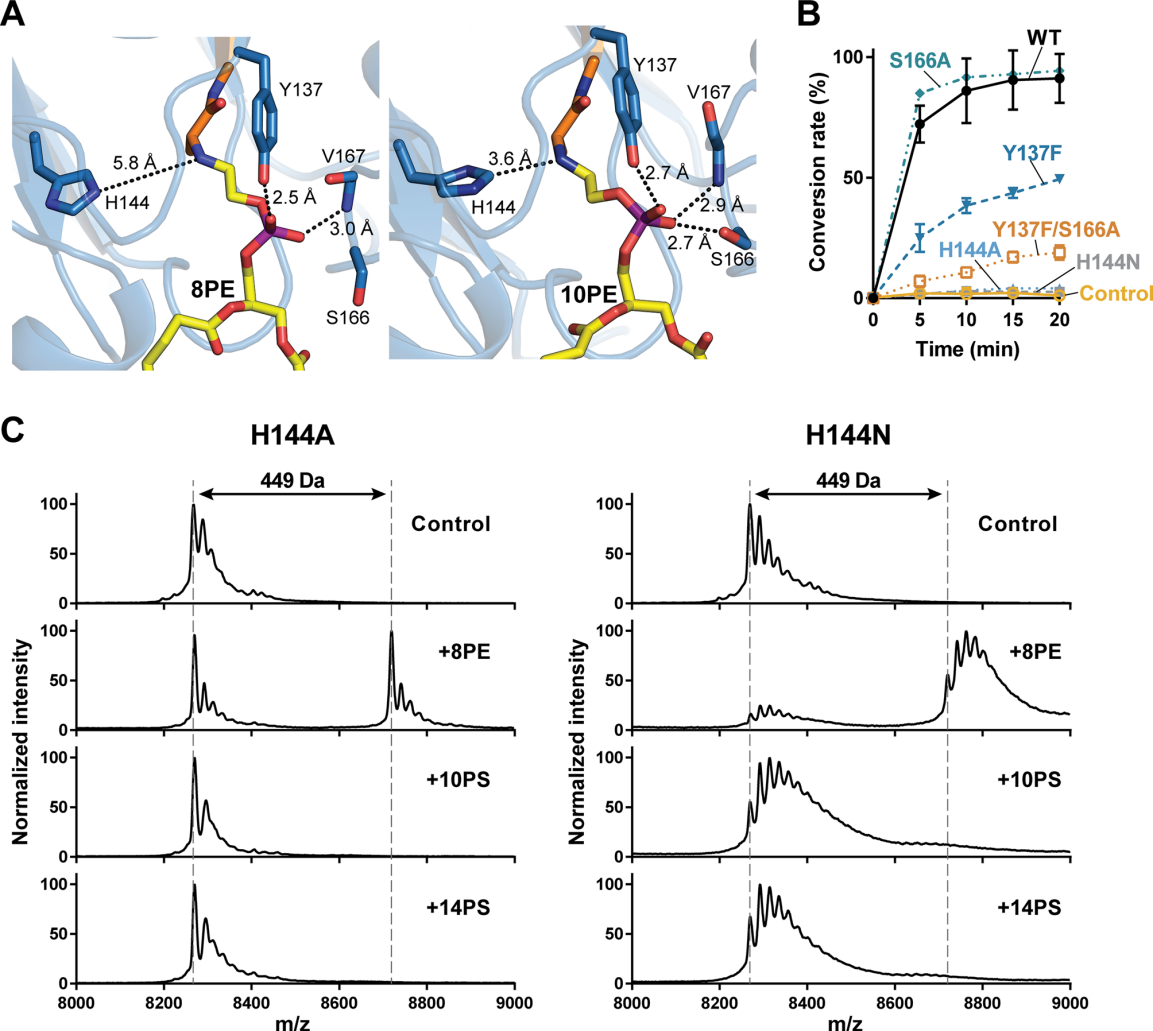
Active site of PS decarboxylation. (**A**) Close-up views of the active site of 8PE-PSD (Left) and 10PE-PSD (Right). Hydrogen bonds and distances between amine group of reduced Schiff base and His-144 are shown in dashed lines with an averaged distance from all protomers in the asymmetric unit. (**B**) LC–MS analysis of the PS decarboxylation activity by the wild type and mutant proteins. Conversion rate was calculated by measuring the relative peak areas of 16:0/18:1 PS and 16:0/18:1 PE. Mean and standard deviation are plotted. Experiments were performed in triplicates. (**C**) Ligand conjugation assay of His-144 mutants. Ligand conjugation by NaCNBH_3_-dependent reduction was performed in H144A (A) or H144N (B) mutants. The conjugated α-chains were analyzed by MALDI-ToF. Theoretical *m/z* values of α-chains are 8261 Da (control), 8712 Da (8PE-conjugated), 8813 Da (10PS-conjugated), and 8925 Da (14PS-conjugated). The MS peaks of control and 8PE-conjugated α-chains are marked as gray dashed lines for comparison of *m/z* values. Theoretical mass difference of control and 8PE-conjugated α-chains is 450 Da.

The phosphate group of PE forms hydrogen bonds with the side chain of Tyr-137 and the backbone amide group of Val-167 in both PE-bound structures, and an additional hydrogen bond is identified with the side chain of Ser-166 in 10PE-PSD (Fig. 5A). The orientation of Pvl-254-linked PE in both structures suggests that His-144 is likely to interact with the departing carboxyl group of PS. To examine whether these amino acid residues are critical for decarboxylation activity, we performed in vitro assays using PSD_1–322_ variants containing a site-specific mutation, followed by LC–MS analysis (Fig. 5B). When Ser-166 was substituted with alanine, however, no significant change from the wild-type protein was observed in the in vitro assay. Meanwhile, Y137F was able to decarboxylate PS at a reduced rate (~ 50%). Tyr-137 is highly conserved among the PSD family (85%) or occasionally replaced by arginine (15%). Interestingly, when both Tyr-137 and Ser-166 were mutated, the double mutant protein Y137F/S166A exhibited a substantially lower activity compared to the wild-type protein, demonstrating the combinatorial effects of these residues in interacting with the phosphate moiety of the lipid substrate. Mutation of the absolutely conserved His-144 completely abolished the decarboxylation activity; neither H144N nor H144A could support the formation of PE in the assay. Considering the importance of His-144, we investigated the specific role of this residue in catalysis by testing whether His-144 mutants were able to form a conjugate product with PS and PE (Fig. 5C). Although the H144A and H144N mutants did not conjugate with 10PS or 14PS, these proteins were able to form an adduct with 8PE, albeit at a reduced efficiency compared to the wild-type protein. Collectively, the data suggest that His-144 mutants were not able to form a stable Schiff base with PS, highlighting the critical role of this residue in correctly recognizing the carboxyl group of substrate lipids.

### Mutagenesis studies on the mechanism of auto-cleavage

**Figure 6:**
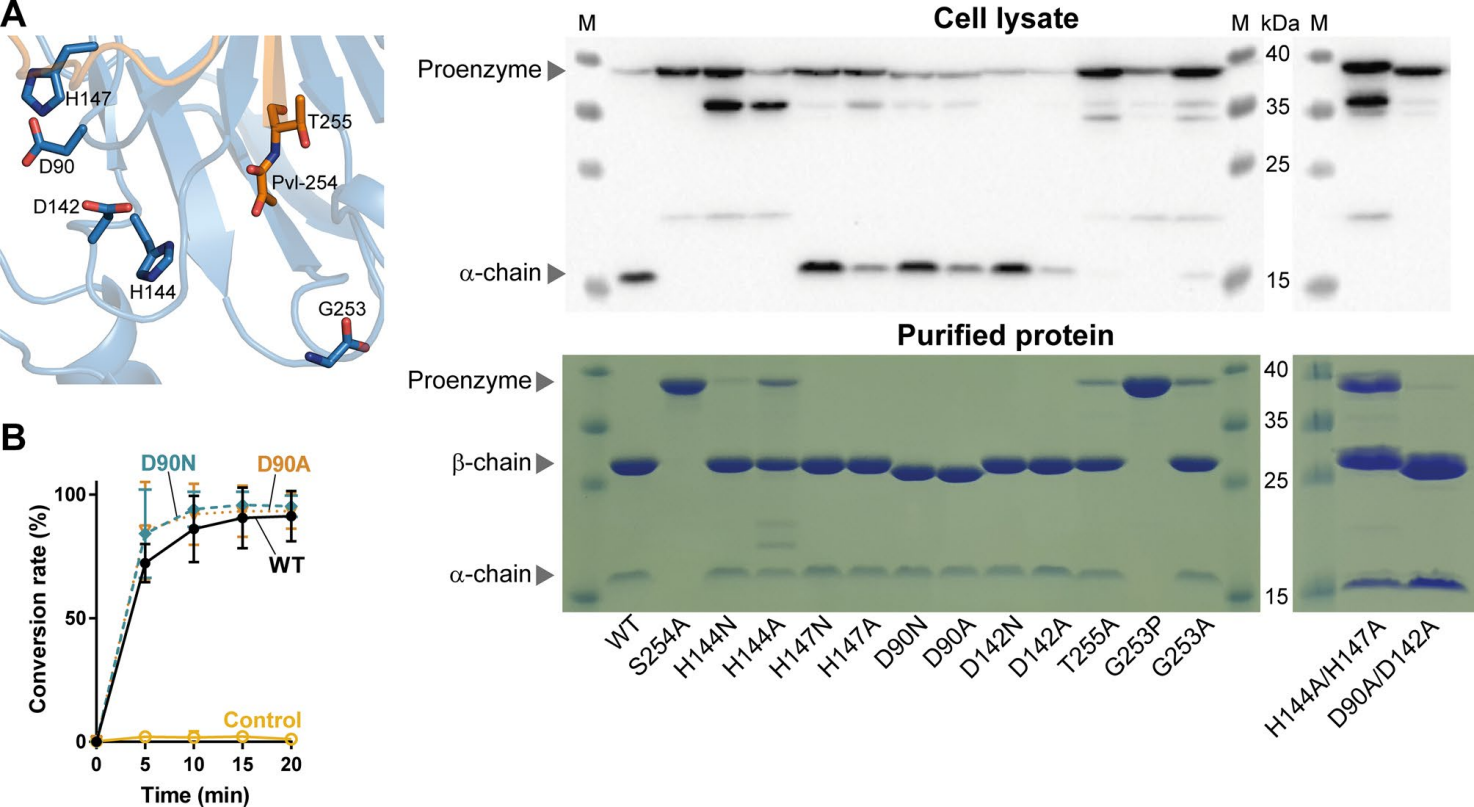
Key residues required for auto-cleavage of proenzyme. (**A**) Putative active site for auto-cleavage is shown where amino acid residues selected for mutagenesis are highlighted as sticks (Left). Wild-type and site-specific mutant proteins are visualized by immunoblot from cell lysates after 4 h induction (Top Right), and the purified recombinant proteins are analyzed by Coomassie staining on 12% SDS-PAGE gel (Bottom Right). Proenzyme (theoretical molecular weight, 36.9 kDa), processed β-chain (28.6 kDa), and α-chain (8.3 kDa). *Lane M* ladder marker. (**B**) LC–MS analysis of PS decarboxylation activity of Asp-90 mutants. Mean and standard deviation are plotted. Experiments were performed in triplicates.

Because PSD is a member of the serine protease family and is known to require a catalytic triad for auto-cleavage^22,23^, we investigated the amino acid residues that are responsible for the maturation of the proenzyme on the basis of genetic and structural information. Ser-308, His-198, and Asp-139 have been reported to organize the catalytic triad of PSD in *P. knowlesi* (corresponding to Ser-254, His-147, and Asp-90 in *E. coli*, respectively); equivalent residues in *S. cerevisiae* are Ser-463, His-345, and Asp-210 (Ser-254, His-144, and Asp-90 in *E. coli*, respectively) (Supplementary Fig. S7)^22,23^. Candidate residues for the maturation of *E. coli* PSD were selected for mutation, and the auto-cleavage of the mutant proteins was analyzed from cell lysates after a 4 h induction period (Fig. 6A**)**. Additionally, we purified the recombinant proteins from cell cultures incubated overnight for induction and analyzed the result of the cleavage, which occurred approximately 24 h after the induction of protein expression had begun**.** Alanine mutation of Ser-254 completely abolished auto-cleavage, which is consistent with a previous report^25^. Similarly, a single mutation of His-144 to alanine or asparagine appeared initially to hamper the auto-proteolysis of the proenzyme; however, both mutants were identified as substantially cleaved in the purified form. An unknown cleavage product with a size slightly smaller than that of the proenzyme was detected from the lysates for both H144A and H144N. However, this unknown fragment was insoluble and not observed after purification. For the H147N and H147A mutants, a significant cleavage occurred after 4 h of induction, which became nearly complete after the purification steps. Similarly, in single mutant proteins of Asp-90, most of the proenzyme was cleaved initially, and the completely processed PSD was identified after purification. However, purified D90N or D90A mutant proteins did not compromise PS decarboxylation activity, unlike His-144 mutants (Fig. 6B). Since the side chains of Asp-90 and His-144 are ~ 9 Å apart in the crystal structures, Asp-90 may not be optimally positioned to depolarize His-144. In the vicinity of the histidine residue, Asp-142 is located at ~ 6 Å; Asp-142 has not been considered as a component of the catalytic triad of PSD in the past. We tested this residue for auto-cleavage by mutating it to asparagine or alanine; however, the results were nearly identical for these mutants and Asp-90 mutants. The results of these single mutants in the pro-enzyme processing suggest that His-144 and His-147 may act as complementary components of the D-H-S triad for *E. coli* PSD, as may Asp-90 and Asp-142. To investigate this possibility, we constructed and analyzed double mutant proteins of these pairs, that is, H144A/H147A and D90A/D142A. Surprisingly, the D90A/D142A double mutant did not undergo auto-cleavage and remained as a proenzyme in the lysates, although became cleaved in the purified form. Meanwhile, the result from H144A/H147A mutant was quite similar to that of H144A; the double mutant was not able to auto-cleave initially, yet displayed significant cleavage products after purification steps. Therefore, our single- and double-mutation experiments support the hypothesis that His-144 plays a major role in activating Ser-254, whereas Asp-90 and Asp-142 can function in complementary to each other in the proenzyme maturation process.

Lastly, we introduced mutations at Gly-253 and Thr-255, which are highly conserved amino acids located next to the nucleophilic Ser-254. Together, these residues comprise the LGST motif, the consensus sequence conserved among the PSD family on which auto-cleavage occurs. G253A, G253P, and T255A could not form auto-cleaved products after 4 h of induction; however, G253A and T255A displayed approximately 70% cleavage after a longer period, as identified in the purified form. G253P remained uncleaved in its purified form, similar to S254A.

## Discussion

**Figure 7:**
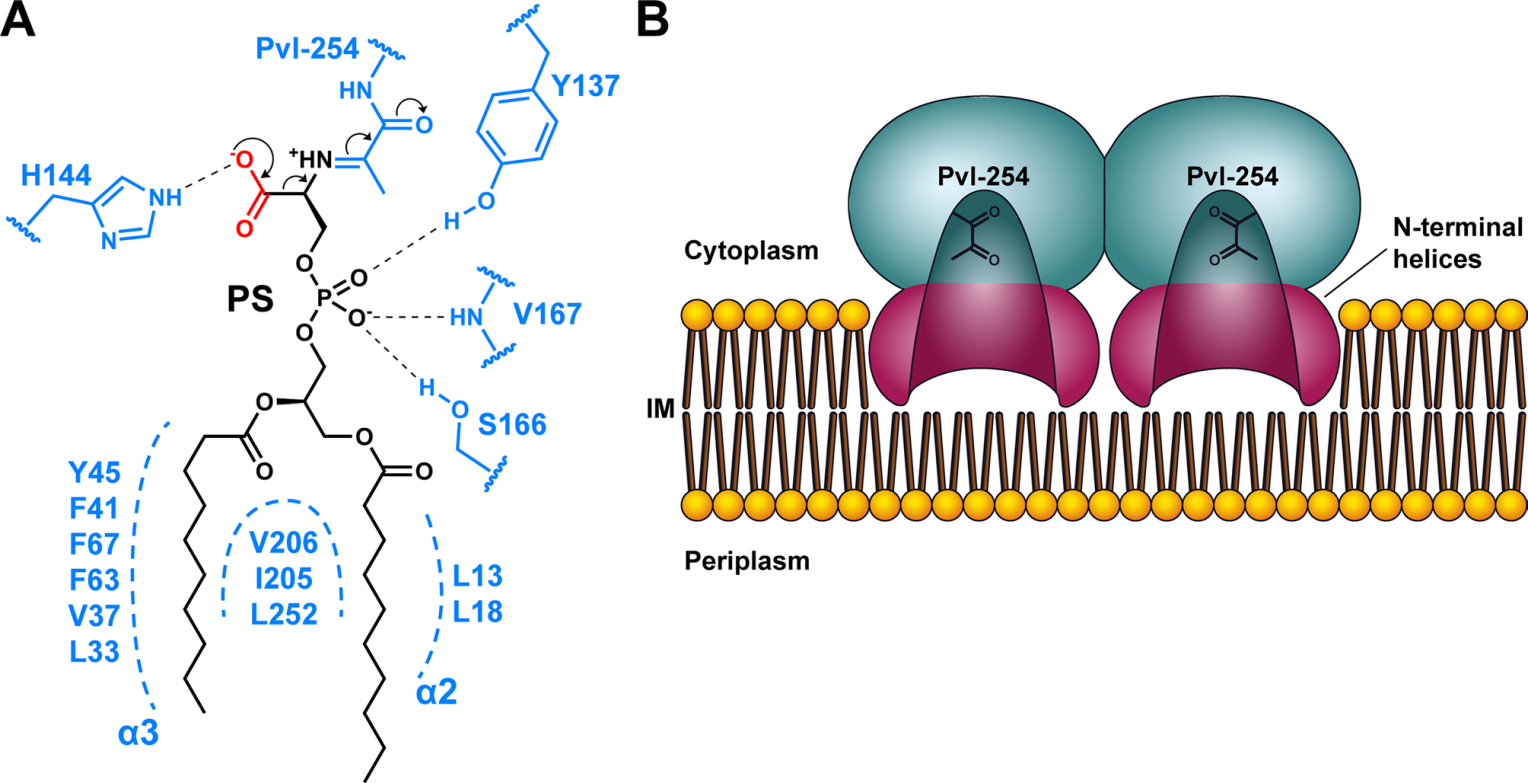
Proposed mechanism of PS decarboxylation and membrane association. (**A**) A scheme of decarboxylation after the formation of a Schiff base intermediate. A 10:0/10:0 acyl-chain of PS is depicted as a representative. Protein residues surrounding the substrate are colored in light blue and carboxylate of PS in red. (**B**) A model of membrane association of *E. coli* PSD, where core domains of PSD αβ-heterodimer are colored in cyan, and N-terminal helices in magenta. Pyruvoyl residues of the active site on each molecule are denoted as black stick. Inner membrane (IM) is shown in orange and brown.

Previous studies have shown that substrate analogs lacking fatty acyl chains, such as serine, phosphoserine, or glycerol phosphoserine were not decarboxylated by PSD, underscoring the importance of essential hydrophobic interactions with fatty acyl chains for substrate binding and catalysis^26^. Our high-resolution structures of PE-bound PSD reveal detailed molecular interactions between the protein and the phospholipid substrate; one fatty acyl chain binds to the larger hydrophobic protein surface defined by Val-37, Phe-41, Phe-63, Phe-67, and Leu-252, whereas the other acyl chain is found on the relatively smaller surface composed of Leu-13, Leu-18, and Thr-204. It is not feasible to unambiguously discriminate the *sn*-isomers of the bound phospholipid, and our structural data suggest that both binding modes may be plausible; that is, the *sn*-1 or *sn*-2 acyl chain is not restricted to binding at a particular site of the enzyme. Although fatty acyl chains are an essential component for effective binding to PSD, it appears that the enzyme mainly recognizes one of the fatty acyl chains via non-specific hydrophobic interactions, whether it is an *sn*-1 or *sn*-2 acyl chain. Phospholipids are highly diverse in length and degree of saturation of fatty acyl chains, where 258 different species have been experimentally identified in *E. coli* to date^27^. As the sole enzyme for synthesizing PE in bacteria, PSD must act promiscuously on a wide range of PS. Current structural data provide insights into the broad specificity of PSD, where the lipid-binding surface permits non-specific hydrophobic interactions with various types of fatty acyl chains of the phospholipid. Additional molecular determinants for PS binding are Tyr-137, His-144, and Ser-166, which interact with phosphate and carboxyl groups. Our structural and biochemical studies clearly support the formation of Schiff base intermediates on the reaction coordinate and suggest that His-144 is likely to interact with the departing carboxyl group of PS, which was also shown to be critical in the formation of a stable Schiff base intermediate (Fig. 7A).

Another valuable feature highlighted in the present structures is that PSD is a monotopic membrane protein that is embedded into a single face of the membrane. Structures of monotopic membrane proteins are extremely rare, accounting for only ~ 0.06% of the total non-redundant structures^28^. We propose that heterotetrameric PSD employs coplanar amphipathic helices in the N-terminus of beta-subunits to associate with the cell membrane, as illustrated in Fig. 7B. Estimation of the protein-membrane border in bacterial cells is further aided by co-crystallized DDM, the length of which roughly encompasses a single membrane leaflet (Supplementary Fig. S8). In this model, the active site is in proximity to the membrane interface, similar to phosphoglycosyl transferase, PglC, another example of monotopic membrane protein^29^.

During the preparation of this manuscript, Watanabe et al*.* reported two crystal structures of *E. coli* PSD in apo and PE-bound states at resolutions of 2.6 and 3.6 Å, respectively^25^. The conclusions drawn by the authors are largely consistent with ours, such as the biological assembly of the enzyme, membrane association of the enzyme, and biochemical properties of key residues contributing to phospholipid recognition. Our high-resolution structures provide more detailed insights into the protein-lipid interactions, indicating the precise active sites on the enzyme where the acyl chains of phospholipids bind. Furthermore, our in-depth analyses of the auto-cleavage of proenzymes required for maturation identified critical residues comprising a D-H-S catalytic triad for *E. coli* PSD; His-144 appears to be the major player in enhancing the nucleophilicity of Ser-254, whereas Asp-90 and Asp-142 can functionally complement to each other during the maturation process. Despite H144A, H144N, H144A/H147, and D90A/D142A were not able to auto-cleave initially, these mutant proteins were eventually cleaved to a certain degree after a longer period, suggesting an alternative mechanism may exist for the activation of the proenzyme. In summary, our data support that the auto-cleavage of the *E. coli* PSD is mainly achieved by D90/D142–H144–S254, analogous to a conventional D-H-S catalytic triad.

The LGST motif, a signature cleavage site conserved among bacterial PSD, is predicted to form a short loop connecting two beta sheets in the proenzyme. It has been proposed that the relief of conformational strain across the cleavage site in proenzymes may be a driving force for auto-cleavage in other pyruvoyl-dependent decarboxylases, including *S*-adenosylmethionine decarboxylase^30^, aspartate decarboxylase^31,32^, and histidine decarboxylase^33^. Our mutagenesis studies showed that G253A, G253P, and T255A exhibited significantly reduced auto-cleavage, suggesting the importance of the correct conformation of the loop and positioning of the serine hydroxyl group for proenzyme maturation. Because of the apparent structural reorganization of the processed α- and β-subunits in the current structures, it is challenging to precisely locate and orient the key residues in the 3-D space prior to the auto-cleavage event. The determination of the pro-PSD structure will provide critical insights regarding the activation process.

The present structural information will be highly valuable in developing a novel class of antibiotics because numerous pathogens are known to require PSD for viability. Additionally, the activity of a mammalian homolog has been reported to be important. In an animal model, mouse embryos lacking *psd1* did not survive past 9 days of development^34^. Human PISD has been associated with various diseases^35^ including cancer^36–38^, Parkinson’s disease^39^, Alzheimer’s disease^40^, liver disease^41^, candidiasis^42–45^, and malaria^46,47^. The physiological significance of PISD has been demonstrated in tumor-initiating cells, where overexpression of PISD downregulates mitochondrial function and inhibits tumor growth^37^. Notably, recent studies demonstrated an emerging role of PISD in tumor regulation, where the tumor repressor LACTB downregulates PISD levels, leading to the alteration of mitochondrial lipid metabolism and differentiation of certain cancer cells^35^. Therefore, the use of therapeutic compounds targeting human PISD may serve as an effective strategy for treating related diseases, including cancer. The structural and functional information from *E. coli* PSD can be extended to understand and predict the biological behaviors of mammalian homologs.

## Methods

### Materials

For membrane protein solubilization, detergent dodecyl b-D-maltopyranoside (DDM) was purchased from Carbosynth, UK (cat. no. DD06199). For conjugation or co-crystallization of phospholipids, 8:0/8:0 PE (8PE; cat. no. 850699), 8:0/8:0 PS (8PS; cat. no. 840031), 10:0/10:0 PS (10PS; cat. no. 840036), 14:0/14:0 PS (14PS; cat. no. 840033), and 16:0/18:1 PS (cat. no. 840034) was purchased from Avanti Polar Lipids, Alabama. Soy PS was purchased from Sigma-Aldrich, Missouri (cat. no. P0474) for functional assay. 2,5-Dihydroxybenzoic acid (2,5-DHB) (part no. 8201346) was purchased from Bruker, Massachusetts for Matrix-Assisted Laser Desorption Ionization (MALDI) analysis.

### Cloning

Plasmid encoding wild type *E. coli psd* gene was purchased from NBRP, Shizuoka, Japan (Resource No. JW4121-AM)^48^. To generate full-length PSD expressing vector, pLATE31-PSD_1–322_, the gene was amplified by the following primers: PSD_ECOLI_1–322_pLATE31 and PSD_ECOLI_1–322_pLATE31 and cloned into pLATE31 vector with a C-terminal His_6_ tag using aLICator LIC Cloning and Expression Kit (Thermo Scientific, Massachusetts). To generate pLATE31-PSD_1–287_ encoding C-terminally truncated *psd* gene with 1–287 residues, DNA fragments were amplified from the pLATE31_PSD_1–322_ by PSD_ECOLI_1–322_pLATE31_For and PSD_ECOLI_1–287_pLATE31_Rev and cloned into the pLATE31 vector. The sequences were verified by DNA sequencing at the Macrogen (Seoul, South Korea). All site-directed point mutations were introduced to full-length *psd* by QuikChange II XL Site-Directed Mutagenesis Kit (Agilent Technologies, California). Primers used in this study are listed in Supplementary table S1.

### Expression and purification

For expression of the PSD_1–287_, *E. coli* BL21(DE3) transformed by the pLATE31-PSD_1–287_ vector was incubated in Luria–Bertani (LB) medium supplemented with 100 μg/mL of ampicillin at 37 °C until OD_600_ = 0.3–0.5. Expression was induced by addition of isopropyl β-d-1-thiogalactopyranoside (IPTG) to a final concentration of 0.1 mM, and the cells were incubated at 20 °C for 19–20 h with shaking at 160 rpm. The cells were harvested by centrifuge at 11,355 rcf (8000 rpm), 4 °C for 10 min, resuspended by buffer A (30 mM Tris–HCl pH 7.5, 150 mM NaCl, and 10% v/v glycerol), and pelleted at 3214 rcf, 4 °C for 30 min. The cell pellets were stored at − 86 °C until used. The cell pellets were lysed by sonication in buffer B [50 mM Tris–HCl pH 7.5, 500 mM NaCl, 10% (v/v) glycerol, and 50 mM imidazole supplemented with 2 mM dithiothreitol (DTT, LPS Solution; Daejeon, South Korea), 1 mM phenylmethylsulfonyl fluoride (PMSF, Sigma-Aldrich) and 1 mg/mL lysozyme (Sigma-Aldrich)]. To solubilize membrane protein, the lysates were incubated with DDM to a final concentration of 1% (w/v) at 4 °C for 1 h with shaking. The solubilized lysates were centrifuged 18,686 rcf (13,000 rpm), 4 °C for 30 min, filtered through 0.2 μm pore size syringe filter, and loaded onto HisTrap HP 5 mL column (GE Healthcare, Illinois) equilibrated with buffer C (50 mM Tris–HCl pH 7.5, 500 mM NaCl, 10% (v/v) glycerol, 50 mM imidazole, and 0.1% (w/v) DDM). The PSD was eluted with 500 mM imidazole gradient in buffer C. The fractions were further purified by size-exclusion chromatography (SEC) on a HiLoad 16/600 Superdex 75 pg column (GE Healthcare) equilibrated with buffer A supplemented with 0.05% (w/v) DDM. The eluted PSD was concentrated using a 3 K MWCO 15 mL or 0.5 mL Amicon Ultra centrifugal filter (Millipore, Massachusetts), flash-frozen in LN2, and stored at -86ºC until used. The wild type and mutants PSD_1–322_ were expressed and purified identically.

For selenomethionine (SeMet) substitution, the *E. coli* BL21(DE3) transformed with pLATE31-PSD_1–287_ vector was grown in M9 media supplemented with 100 μg/mL ampicillin at 37 °C to OD_600_ = 0.2. Methionine biosynthesis was inhibited by the addition of lysine, phenylalanine, and threonine at 100 mg/L, isoleucine, leucine, valine, and SeMet at 50 mg/L^49^. Expression was induced by 0.1 mM IPTG 15 min after the addition of the amino acids. The cells were incubated and harvested as that of the native protein. The SeMet substituted PSD was purified similarly to that of the native protein with minor changes. Briefly, all buffers used in purification were degassed. The cells were lysed by sonication in buffer B except DTT replaced by 5 mM tris(2-carboxyethyl)phosphine (TCEP, Sigma-Aldrich). On the affinity purification and SEC, buffers were additionally supplemented with 0.2 mM TCEP.

### Ligand conjugation by reduction for structural characterization

Schiff base reduction was performed as described by Li and Dowhan^19^ with minor changes. Prior to the reduction, purified PSD_1–287_ was buffer exchanged by SEC on a HiLoad 16/600 Superdex 75 pg column to buffer D (30 mM HEPES pH 7.5, 150 mM NaCl, 10% (v/v) glycerol, and 0.05% (w/v) DDM). To conjugate the ligands to the protein, 2.4–2.5 mg of 38.7 μM PSD_1–287_, 5 M NaCNBH_3_, and 10 mM 8PE/10PS/14PS were sequentially mixed to a final concentration of 34.4 μM, 50 mM, and 1 mM, respectively while vortexing. For full-length PSD, 3.7 mg of 25.3 μM PSD_1–322_ were used to a final concentration of 22.5 μM. The mixture was incubated in a water bath at 37 °C for 10 h (PSD_1–322_) or 14 h (PSD_1–287_), and buffer exchanged by a 3 K MWCO 15 mL Amicon Ultra centrifugal filter to the buffer A supplemented with 0.05% (w/v) DDM. The conjugation efficiency was estimated by MALDI analysis.

### Crystallization and structural determination

Concentrated protein solutions were cleared by centrifugation at 17,000 rcf, 4 °C for 10 min prior to crystallization. The crystallization was performed by sitting drop vapor diffusion method at 20 °C by mixing a 0.8 μL of protein solution with 0.8 μL of reservoir solution equilibrating against 60 μL of the reservoir solution. Rod-shaped Apo-PSD1 crystallized at a concentration of 8.5 mg/mL PSD_1–287_ within one week in 0.1 M Bis–Tris pH 6.3, 28% (w/v) polyethylene glycol monomethyl ether (PEGMME) 2000, and 5% (w/v) 1,6-hexanediol. For rhombus-shaped Apo-PSD2, 10 mg/mL PSD_1–287_ were mixed with 50 mM 8PS with a 9:1 (v/v) ratio, incubated on ice for 10 min, cleared by centrifugation at 17,000 rcf, 4 °C for 10 min, and used directly to crystallization. The crystals were formed within one week in 0.18 M ammonium acetate, 0.09 M Tris pH 8.5, 22.5% (w/v) polyethylene glycol (PEG) 3350, and 5% (v/v) Jeffamine M-600 pH 7.0. However, there was no measurable electron density of the lipid. 8PE-bound PSD_1–287_ crystals were formed at a concentration of 8.0 mg/mL of 8PE-conjugated PSD_1–287_ prepared above in 0.1 M potassium thiocyanate and 30% (w/v) PEGMME 2000. 10:0/10:0 PE(10PE)-bound PSD_1–287_ crystals were observed at a concentration of 8.8 mg/mL of 10PS-conjugated PSD_1–287_ in 0.2 M lithium sulfate monohydrate, 0.1 M Tris pH 8.5, 25% (w/v) PEG 3350. SeMet substituted Apo-PSD1 crystals were formed with rod-shaped at a concentration of 9.7 mg/mL in the identical condition to that of the unlabeled sample.

All crystals were cryoprotected by mother liquor supplemented with 20% (v/v) glycerol and flash-frozen in liquid nitrogen. X-ray diffraction data were collected under cryogenic conditions using the ADSC Q270 detector at Pohang Accelerator Laboratory (PAL; Pohang, South Korea) beamline 7A (SeMet, Apo-PSD1 and 2) or using Eiger X 9 M detector at PAL beamline 5C (8PE- and 10PE-bound PSD_1–287_) (Table S1). SeMet data were indexed, integrated, scaled by HKL2000^50^. All native data were processed by XDS^51^ and merged by Aimless^52^ in CCP4 suite^53^. To solve the phase problem, the processed SeMet data were used for Single-wavelength Anomalous Dispersion (SAD) phasing by AutoSol^54^ in Phenix suite^55^. Apo-PSD1 structure was built by iterative runs of real space model building using Coot^56^, and data refinement by REFMAC5^57^, or Phenix Refine^58^. The Apo-PSD1 structure was used as a search model of molecular replacement for determining other structures. After the main chains were built, pyruvoyl residues were modeled. Newly introduced pyruvate residues are linked with Thr-255 using JLigand^59^. To model 8PE- and 10PE-conjugated pyruvoyl residue, ACEDRG^60^ was used to generate a molecular model and define structural restraints of lipid-conjugated pyruvate. PyMOL^61^ was used to visualize the structures.

### Multi-angle light scattering (MALS) analysis

Purified *E. coli* PSD_1–287_ were buffer exchanged to buffer E (30 mM Tris–HCl pH 7.5, 150 mM NaCl, supplemented with 0.05% (w/v) DDM), concentrated to 2.2 mg/mL, flash-frozen in LN2, and stored at − 86 °C until used. The concentrated protein was separated by Superdex 200 10/300 GL equilibrated with the degassed buffer E with a flow rate of 0.5 mL/min. The eluents were analyzed by MALS detector (Dawn Heleos II, Wyatt) equipped with differential refractive index (dRI; Optilab T-Rex, Wyatt) and UV detector. The signals were analyzed by ASTRA 6 program. Theoretical molecular weight and extinction coefficient of the PSD_1–287_ used for the calculation are 33,085.29 Da and 46,410 /M/cm, respectively.

### Membrane association assay

*E. coli* BL21(DE3) cells containing wild-type PSD_1–322_ (WT) or truncation variants (ΔH1, 13–322; ΔH2, 30–322; ΔH3, 46–322) expressing plasmids were incubated in 1 L of LB medium supplemented with 100 μg/mL of ampicillin. When OD_600_ reaches ~ 0.1, the protein expressions were induced by addition of IPTG to a final concentration of 0.1 mM. The flask containing the expression cell was incubated either at 20 °C (WT, ΔH2, and ΔH3) or 37 °C (ΔH1) until OD_600_ reaches 1.0–1.5 shaking at 160 rpm. The cells were harvested and stored as described above. The cell pellets were lysed in 1 × PBS (3 mM Na_2_HPO_4_, 1.1 mM KH_2_PO_4_, and 160 mM NaCl, pH 7.4; LPS Solution, Republic of Korea) and sonicated. Inclusion body was separated by centrifugation at 21,672 rcf (14,000 rpm) and lysed in 25 mL of 1 × PBS. The supernatant was applied to ultracentrifuge at 120,000 rcf (34,200 rpm, Ti 70 rotor equipped with Optima L-100K, Beckman Coulter, USA) for 1 h. After the ultracentrifuge, the supernatant (soluble fraction) and the pellet (membrane fraction) were collected. The pellet was lysed in 25 mL of 1 × PBS. Each fraction was analyzed by 12% SDS-PAGE and visualized by western blotting using HRP Anti-6 × His tag antibody (cat. no. ab1187, Abcam, UK). Original images of the membrane containing blots are shown in Supplementary Fig. S9.

### LC–MS analysis of functional activity

The enzymatic reaction was initiated by mixing PSD_1–287_ and soy PS in buffer A supplemented with 0.05% (w/v) DDM to a final concentration of 10 μM and 1 mM, respectively, and incubated in a water bath at 37 °C for 30 min. The reaction mixtures were quenched by adding an equal volume of 1 M HCl. Subsequently, lipids in the reaction samples were extracted by Folch’s method^62^. Briefly, one volume of the quenched mixtures was transferred to 5 volumes of the chloroform/methanol (2:1, v/v) in a glass tube. The tube vortexed and centrifuged briefly at 500 rcf, 4 °C for 10 min. By carefully discarding the upper phase, the lower phase was collected in a new glass tube and dried out. The extracted lipids were dissolved in methanol and subjected to LC–MS analysis. An aliquot of 20 μL of the sample was injected into a reversed phase HPLC column (ZORBAX RR Eclipse Plus C18, 95 Å, 4.6 × 100 mm, 3.5 µm; Agilent Technologies) attached to Agilent 1260 Infinity Quaternary LC system (Agilent Technologies). Chromatographic separation was performed by using Solvent A (10 mM ammonium acetate in acetonitrile:water 60:40 (v/v)), and solvent B [10 mM ammonium acetate in isopropanol:acetonitrile 90:10 (v/v)], using a gradient step with the flow rate of 0.8 mL/min as following: (1) 70% B for 1 min (column equilibration); (2) a linear gradient from 70 to 100% B for 11 min; (3) a hold at 100% B for 1 min (column wash); (4) a linear gradient from 100 to 70% B for 1 min; (5) a hold at 70% B for 1 min (column equilibration) with additional hold for 5 min using Post time. Eluted fractions were loaded to the MS and MS/MS system (Agilent 6520 Q-TOF LC/MS; Agilent Technologies) and analyzed in the positive ion mode utilizing Dual ESI as an ionization source. Major phospholipids were identified and relatively quantified from MS and MS/MS spectra by the MassHunter program with the aid of LipidBLAST^63^. Results were visualized by GraphPad Prism^64^. Functional assay of the PSD_1–322_ was performed in the same manner as the PSD_1–287_ with final enzyme concentrations for the reaction reduced to 1 μM. For conversion rate analysis of wild type and mutants PSD_1–322_, 1 μM of the enzyme is incubated with 1 mM 16:0/18:1 PS in a 37 °C water bath for 0, 5, 10, 15, and 20 min. Following extraction of phospholipids and LC–MS analysis were performed as described above.

### Ligand conjugation by reduction of His-144 mutants

Conjugation assay was performed similarly as used for the structure characterization described above with minor changes. Buffer exchanged PSD_1–322_ variants (H144A and H144N) were concentrated to 0.431 mg/mL (H144A) or 0.901 mg/mL (H144N). The protein, 5 M NaCNBH_3_, and 10 mM 8PE/10PS/14PS were sequentially mixed with a volume ratio of 89:1:10 while vortexing. The mixture was incubated in a water bath at 37 °C for 15 h and analyzed by MALDI-ToF.

### MALDI-ToF for conjugation efficiency analysis

For preparation of matrix solution, 2,5-DHB was dissolved in acetonitrile/0.1% (v/v) TFA (30:70, v/v; TA30) to a final concentration of 20 mg/mL. Approximately 1 mg/mL purified protein samples were tenfold diluted by adding 0.1% (v/v) trifluoroacetic acid (TFA). The diluted solution was mixed with an equal volume of the matrix solution. Then, a 0.5 μL of the mixture was spotted to MTP 384 ground steel BC targets. The target plate was loaded to autoflex speed TOF/TOF (Bruker). Measurements were conducted on linear positive ion mode using flexControl program at laser frequency of 2000 Hz. For each dataset, 2000 shots were collected by random walking and merged.

### Auto-activation assay

*E. coli* cells expressing wild-type or site-specific variants were incubated in a 20 mL LB media with 100 μg/mL ampicillin and induced when OD_600_ is 0.4–0.6 and incubated for 4 h at 20 °C. The cells were harvested by centrifugation at 4 °C, 3214 rcf (3900 rpm). The pellets were lysed in 700 μL of 1 × PBS and analyzed by 12% SDS-PAGE and western blotting with the anti-histag antibody. For the assay of the purified proteins, the expression of the protein was induced at 20 °C for 19–20 h. After the cell harvest, the protein undergoes purification process at room temperature which usually took ~ 24 h. The purified proteins were analyzed by 12% SDS-PAGE and stained by Coomassie blue.

## Supplementary Information

Supplementary Information.

## Data Availability

The atomic coordinates and structure factors for Apo-PSD1, Apo-PSD2, 8PE-PSD, and 10PE-PSD are deposited to Protein Data Bank under accession code 7CNW, 7CNX, 7CNY, and 7CNZ, respectively.

## References

[1] Sohlenkamp C, Geiger O (2015). Bacterial membrane lipids: Diversity in structures and pathways. FEMS Microbiol. Rev..

[2] Vance JE, Tasseva G (2013). Formation and function of phosphatidylserine and phosphatidylethanolamine in mammalian cells. Biochim. Biophys. Acta Mol. Cell Biol. Lipids.

[3] Borkenhagen LF, Kennedy EP, Fielding L (1961). Enzymatic formation and decarboxylation of phosphatidylserine. J. Biol. Chem..

[4] Cronan JE, Vagelos PR (1972). Metabolism and function of the membrane phospholipids of *Escherichia coli*. BBA Rev. Biomembr..

[5] Bell RM, Mavis RD, Osborn MJ, Roy Vagelos P (1971). Enzymes of phospholipid metabolism: Localization in the cytoplasmic and outer membrane of the cell envelope of *Escherichia**coli* and *Salmonella**typhimurium*. BBA Biomembr..

[6] Di Bartolomeo F, Wagner A, Daum G (2017). Cell biology, physiology and enzymology of phosphatidylserine decarboxylase. Biochim. Biophys. Acta Mol. Cell Biol. Lipids.

[7] Denkins YM, Schroit AJ (1986). Phosphatidylserine decarboxylase: Generation of asymmetric vesicles and determination of the transbilayer distribution of fluorescent phosphatidylserine in model membrane systems. BBA Biomembr..

[8] Drechsler C (2018). Preparation of asymmetric liposomes using a phosphatidylserine decarboxylase. Biophys. J..

[9] McMahon HT, Boucrot E (2015). Membrane curvature at a glance. J. Cell Sci..

[10] Bigay J, Antonny B (2012). Curvature, lipid packing, and electrostatics of membrane organelles: Defining cellular territories in determining specificity. Dev. Cell.

[11] Vitrac H, MacLean DM, Jayaraman V, Bogdanov M, Dowhan W (2015). Dynamic membrane protein topological switching upon changes in phospholipid environment. Proc. Natl. Acad. Sci. U.S.A..

[12] Luo H, Lin Y, Gao F, Zhang CT, Zhang R (2014). DEG 10, an update of the database of essential genes that includes both protein-coding genes and noncoding genomic elements. Nucleic Acids Res..

[13] Leventis PA, Grinstein S (2010). The distribution and function of phosphatidylserine in cellular membranes. Annu. Rev. Biophys..

[14] Zborowski J, Dygas A, Wojtczak L (1983). Phosphatidylserine decarboxylase is located on the external side of the inner mitochondrial membrane. FEBS Lett..

[15] Bleijerveld OB, Brouwers JFHM, Vaandrager AB, Helms JB, Houweling M (2007). The CDP-ethanolamine pathway and phosphatidylserine decarboxylation generate different phosphatidylethanolamine molecular species. J. Biol. Chem..

[16] Heikinheimo L, Somerharju P (1998). Preferential decarboxylation of hydrophilic phosphatidylserine species in cultured cells. Implications on the mechanism of transport to mitochondria and cellular aminophospholipid species compositions. J. Biol. Chem..

[17] Bürgermeister M, Birner-Grünberger R, Nebauer R, Daum G (2004). Contribution of different pathways to the supply of phosphatidylethanolamine and phosphatidylcholine to mitochondrial membranes of the yeast *Saccharomyces**cerevisiae*. Biochim. Biophys. Acta Mol. Cell Biol. Lipids.

[18] Calzada E (2019). Phosphatidylethanolamine made in the inner mitochondrial membrane is essential for yeast cytochrome bc 1 complex function. Nat. Commun..

[19] Li, Q. X. & Dowhan, W. Structural characterization of Escherichiacoli phosphatidylserine decarboxylase. *J. Biol. Chem.* **263**, 11516 (1988). 3042771

[20] Satre, M. & Kennedy, E. P. Identification of bound pyruvate essential for the activity of phosphatidylserine decarboxylase of Escherichiacoli. *J. Biol. Chem.* **253**, 479–483 (1978). 338609

[21] Li, Q. X. & Dowhan, W. Studies on the mechanism of formation of the pyruvate prosthetic group of phosphatidylserine decarboxylase from Escherichiacoli. *J. Biol. Chem.* **265**, 4111–4115 (1990). 2406271

[22] Choi, J. Y., Duraisingh, M. T., Marti, M., Ben Mamoun, C. & Voelker, D. R. From protease to decarboxylase: The molecular metamorphosis of phosphatidylserine decarboxylase. *J. Biol. Chem.*10.1074/jbc.M115.642413 (2015). 10.1074/jbc.M115.642413 PMC4409258 25724650

[23] Ogunbona, O. B., Onguka, O., Calzada, E. & Claypool, S. M. Multitiered and cooperative surveillance of mitochondrial phosphatidylserine decarboxylase 1. *Mol. Cell. Biol.*10.1128/mcb.00049-17 (2017). 10.1128/MCB.00049-17 PMC5559681 28606933

[24] Voelker, D. R. Phosphatidylserine decarboxylase. *Biochim. Biophys. Acta Lipids Lipid Metab.* **1348**, 236–244 (1997). 10.1016/s0005-2760(97)00101-x 9370338

[25] Watanabe, Y., Watanabe, Y. & Watanabe, S. Structural basis for phosphatidylethanolamine biosynthesis by bacterial phosphatidylserine decarboxylase. *Structure*. 10.1016/j.str.2020.04.006 (2020). 10.1016/j.str.2020.04.006 32402247

[26] Dowhan, W., Wickner, W. T. & Kennedy, E. P. Purification and properties of phosphatidylserine decarboxylase from Escherichiacoli. *J. Biol. Chem.* **249**, 3079–3084 (1974). 4598120

[27] Sajed, T. *et al.* ECMDB 2.0: A richer resource for understanding the biochemistry of E. coli. *Nucleic Acids Res.*10.1093/nar/gkv1060 (2016). 10.1093/nar/gkv1060 PMC4702790 26481353

[28] Allen, K. N., Entova, S., Ray, L. C. & Imperiali, B. Monotopic membrane proteins join the fold. *Trends Biochem. Sci.* **44**, 7–20 (2019). 10.1016/j.tibs.2018.09.013 PMC6309722 30337134

[29] Ray, L. C. *et al.* Membrane association of monotopic phosphoglycosyl transferase underpins function. *Nat. Chem. Biol.* **14**, 528–541 (2018). 10.1038/s41589-018-0054-z PMC6202225 29769739

[30] Tolbert, W. D. *et al.* Mechanism of human S-adenosylmethionine decarboxylase proenzyme processing as revealed by the structure of the S68A mutant. *Biochemistry*. 10.1021/bi0268854 (2003). 10.1021/bi0268854 12600205

[31] Albert, A. *et al.* Crystal structure of aspartate decarboxylase at 2.2 Å resolution provides evidence for an ester in protein self-processing. *Nat. Struct. Biol.*10.1038/nsb0498-289 (1998). 10.1038/nsb0498-289 9546220

[32] Schmitzberger, F. *et al.* Structural constraints on protein self-processing in l-aspartate-α-decarboxylase. *EMBO J.*10.1093/emboj/cdg575 (2003). 10.1093/emboj/cdg575 PMC291833 14633979

[33] Gallagher, T., Rozwarski, D. A., Ernst, S. R. & Hackert, M. L. Refined structure of the pyruvoyl-dependent histidine decarboxylase from Lactobacillus 30a. *J. Mol. Biol.*10.1006/jmbi.1993.1168 (1993). 10.1006/jmbi.1993.1168 8464063

[34] Steenbergen, R. *et al.* Disruption of the phosphatidylserine decarboxylase gene in mice causes embryonic lethality and mitochondrial defects. *J. Biol. Chem.* **280**, 40032–40040 (2005). 10.1074/jbc.M506510200 PMC2888304 16192276

[35] Calzada, E., Onguka, O. & Claypool, S. M. Phosphatidylethanolamine Metabolism in Health and Disease. *International Review of Cell and Molecular Biology* Vol. 321 (Elsevier, 2016). 10.1016/bs.ircmb.2015.10.001 PMC4778737 26811286

[36] Keckesova, Z. *et al.* LACTB is a tumour suppressor that modulates lipid metabolism and cell state. *Nature.*10.1038/nature21408 (2017). 10.1038/nature21408 PMC6246920 28329758

[37] Chidley, C., Trauger, S. A., Birsoy, K. & O’Shea, E. K. The anticancer natural product ophiobolin A induces cytotoxicity by covalent modification of phosphatidylethanolamine. *Elife.*10.7554/eLife.14601 (2016). 10.7554/eLife.14601 PMC4942256 27403889

[38] Chen, Y. C. *et al.* Functional isolation of tumor-initiating cells using microfluidic-based migration identifies phosphatidylserine decarboxylase as a key regulator. *Sci. Rep.*10.1038/s41598-017-18610-5 (2018). 10.1038/s41598-017-18610-5 PMC5762897 29321615

[39] Wang, S. *et al.* Phosphatidylethanolamine deficiency disrupts α-synuclein homeostasis in yeast and worm models of Parkinson disease. *Proc. Natl. Acad. Sci. U.S.A.*10.1073/pnas.1411694111 (2014). 10.1073/pnas.1411694111 PMC4183298 25201965

[40] Nesic, I. *et al.* Alterations in phosphatidylethanolamine levels affect the generation of Aβ. *Aging Cell.*10.1111/j.1474-9726.2011.00760.x (2012). 10.1111/j.1474-9726.2011.00760.x 22023223

[41] Van Der Veen, J. N., Lingrell, S., Da Silva, R. P., Jacobs, R. L. & Vance, D. E. The concentration of phosphatidylethanolamine in mitochondria can modulate ATP production and Glucose Metabolism in Mice. *Diabetes.*10.2337/db13-0993 (2014). 10.2337/db13-0993 24677714

[42] Chen, Y. L. *et al.* Phosphatidylserine synthase and phosphatidylserine decarboxylase are essential for cell wall integrity and virulence in Candidaalbicans. *Mol. Microbiol.*10.1111/j.1365-2958.2009.07018.x (2010). 10.1111/j.1365-2958.2009.07018.x 20132453

[43] Khandelwal, N. K., Sarkar, P., Gaur, N. A., Chattopadhyay, A. & Prasad, R. Phosphatidylserine decarboxylase governs plasma membrane fluidity and impacts drug susceptibilities of Candidaalbicans cells. *Biochim. Biophys. Acta Biomembr.*10.1016/j.bbamem.2018.05.016 (2018). 10.1016/j.bbamem.2018.05.016 29856993

[44] Davis, S. E. *et al.* Candidaalbicans cannot acquire sufficient ethanolamine from the host to support virulence in the absence of de novo phosphatidylethanolamine synthesis. *Infect. Immun.*10.1128/IAI.00815-17 (2018). 10.1128/IAI.00815-17 PMC6056879 29866908

[45] Cassilly, C. D. & Reynolds, T. B. PS, it’s complicated: The roles of phosphatidylserine and phosphatidylethanolamine in the pathogenesis of Candidaalbicans and other microbial pathogens. *J. Fungi.*10.3390/jof4010028 (2018). 10.3390/jof4010028 PMC5872331 29461490

[46] Choi, J. Y. *et al.* Characterization of plasmodium phosphatidylserine decarboxylase expressed in yeast and application for inhibitor screening. *Mol. Microbiol.*10.1111/mmi.13280 (2016). 10.1111/mmi.13280 PMC4898484 26585333

[47] Roggero, R. *et al.* Unraveling the mode of action of the antimalarial choline analog G25 in Plasmodiumfalciparum and Saccharomycescerevisiae. *Antimicrob. Agents Chemother.*10.1128/AAC.48.8.2816-2824.2004 (2004). 10.1128/AAC.48.8.2816-2824.2004 PMC478495 15273086

[48] Kitagawa M (2005). Complete set of ORF clones of *Escherichia**coli* ASKA library (A complete set of *E. coli* K-12 ORF archive): Unique resources for biological research. DNA Res..

[49] Doublié, S. Production of selenomethionyl proteins in prokaryotic and eukaryotic expression systems. in *Macromolecular Crystallography Protocols* 91–108 (2007). 10.1007/978-1-59745-209-0_5. 10.1007/978-1-59745-209-0_5 17272838

[50] Otwinowski Z, Minor W (1997). Processing of X-ray diffraction data collected in oscillation mode. Methods Enzymol..

[51] Kabsch W (2010). XDS. Acta Crystallogr. Sect. D Biol. Crystallogr..

[52] Evans PR, Murshudov GN (2013). How good are my data and what is the resolution?. Acta Crystallogr. Sect. D Biol. Crystallogr..

[53] Winn MD (2011). Overview of the CCP4 suite and current developments. Acta Crystallogr. D Biol. Crystallogr..

[54] Terwilliger TC (2009). Decision-making in structure solution using Bayesian estimates of map quality: The PHENIX AutoSol wizard. Acta Crystallogr. Sect. D Biol. Crystallogr..

[55] Liebschner D (2019). Macromolecular structure determination using X-rays, neutrons and electrons: Recent developments in Phenix. Acta Crystallogr. Sect. D Struct. Biol..

[56] Emsley P, Lohkamp B, Scott WG, Cowtan K (2010). Features and development of Coot. Acta Crystallogr. Sect. D Biol. Crystallogr..

[57] Murshudov GN (2011). REFMAC5 for the refinement of macromolecular crystal structures. Acta Crystallogr. Sect. D Biol. Crystallogr..

[58] Afonine PV (2012). Towards automated crystallographic structure refinement with phenix.refine. Acta Crystallogr. Sect. D Biol. Crystallogr..

[59] Lebedev AA (2012). JLigand: A graphical tool for the CCP4 template-restraint library. Acta Crystallogr. Sect. D Biol. Crystallogr..

[60] Long F (2017). AceDRG: A stereochemical description generator for ligands. Acta Crystallogr. Sect. D Struct. Biol..

[61] Schrödinger L (2015). The PyMol Molecular Graphics System, Versión 1.8. Thomas Holder.

[62] Folch J, Lees M, Sloane Stanley GH (1957). A simple method for the isolation and purification of total lipids from animal tissues. J. Biol. Chem..

[63] Kind T (2013). LipidBlast in silico tandem mass spectrometry database for lipid identification. Nat. Methods.

[64] One-way ANOVA with Dunnett's post test was performed using GraphPad Prism version 7 for Windows, GraphPad Software, La Jolla California USA, www.graphpad.com.

