# Author Correction: Structural insights into phosphatidylethanolamine formation in bacterial membrane biogenesis

**DOI:** 10.1038/s41598-021-92397-4

**Published:** 2021-06-15

**Authors:** Gyuhyeok Cho, Eunju Lee, Jungwook Kim

**Affiliations:** Department of Chemistry, Gwangju Institute of Science and Technology, Gwangju, 61005 Republic of Korea

Correction to: *Scientific Reports* 10.1038/s41598-021-85195-5, published online 11 March 2021

The original version of this Article omitted the Funding section. The Funding section now reads:

“This work was supported by GIST Research Institute (GRI) grant funded by the GIST.”

The original Article has been corrected.

