# Supplementary material for: Structural insights into phosphatidylethanolamine formation in bacterial membrane biogenesis
Source: Sci Rep. 2021 Mar 11;11:5785. doi: 10.1038/s41598-021-85195-5 (PMC7952604; doi:10.1038/s41598-021-85195-5)
Supplement: Supplementary file 1 — Supplementary Information. [file 41598_2021_85195_MOESM1_ESM.pdf]

Supplementary information for

Structural insights into phosphatidylethanolamine formation in bacterial membrane biogenesis

Gyuhyeok Cho[1], Eunju Lee[1], and Jungwook Kim[1*]

[1]Department of Chemistry, Gwangju Institute of Science and Technology, Gwangju, 61005, Republic of Korea

[*]E-mail: jwkim@gist.ac.kr

This PDF file includes:

Supplementary Figure S1 to S9

Supplementary Table S1

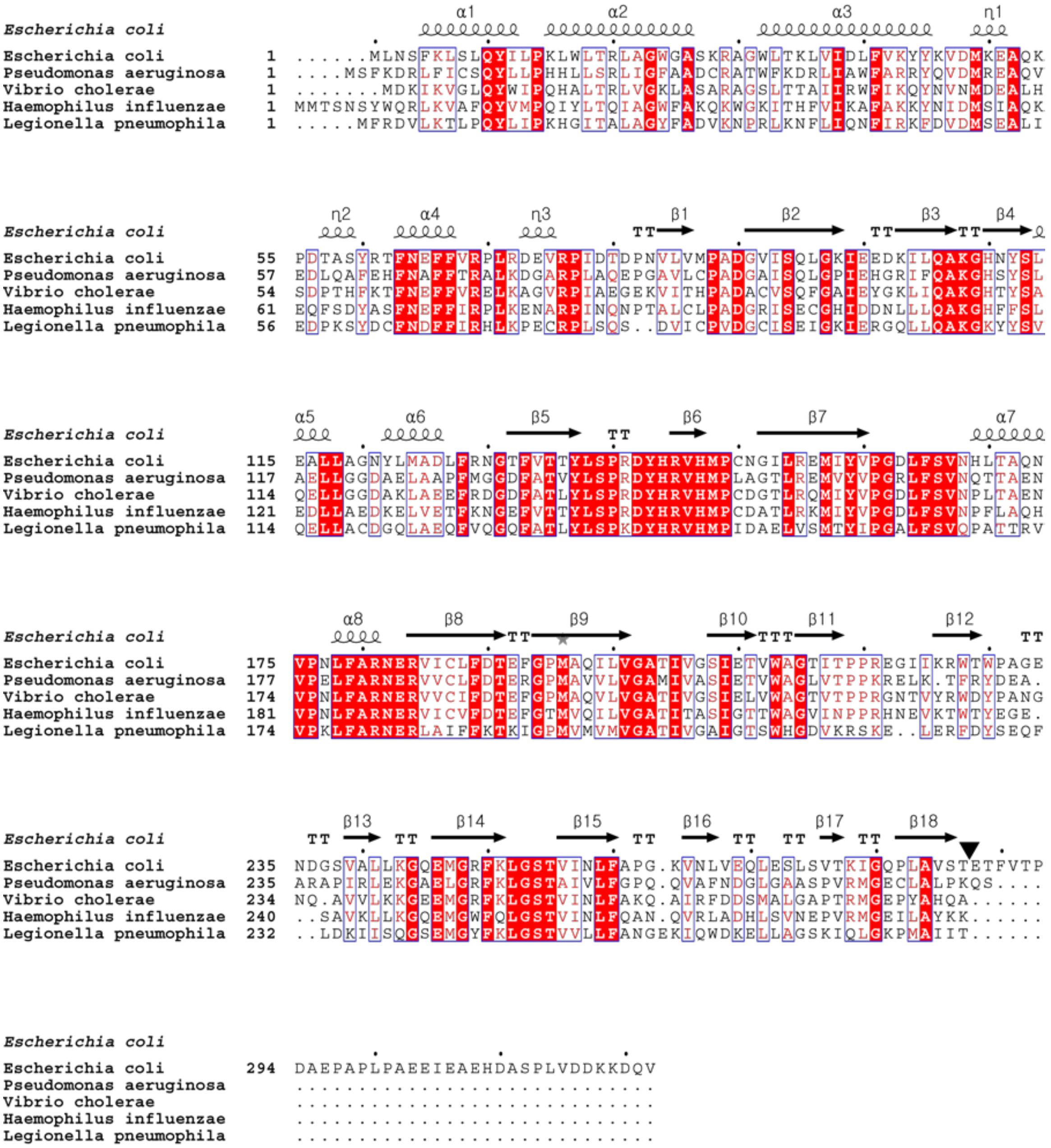

Supplementary Figure S1. Multiple sequence alignment of PSD from Gammaproteobacteria. Sequences of five PSD (UniProt accession ID: PSD_ECOLI, PSD_PSEAE, PSD_VIBCH, PSD_HAEIN, and PSD_LEGPH) in various orders of the class are aligned by Clustal Omega[1]. Secondary structures from *E. coli* PSD are mapped above the alignment by ESPript - http://espript.ibcp.fr[2]. Truncated site for crystallization is designated as black triangle.

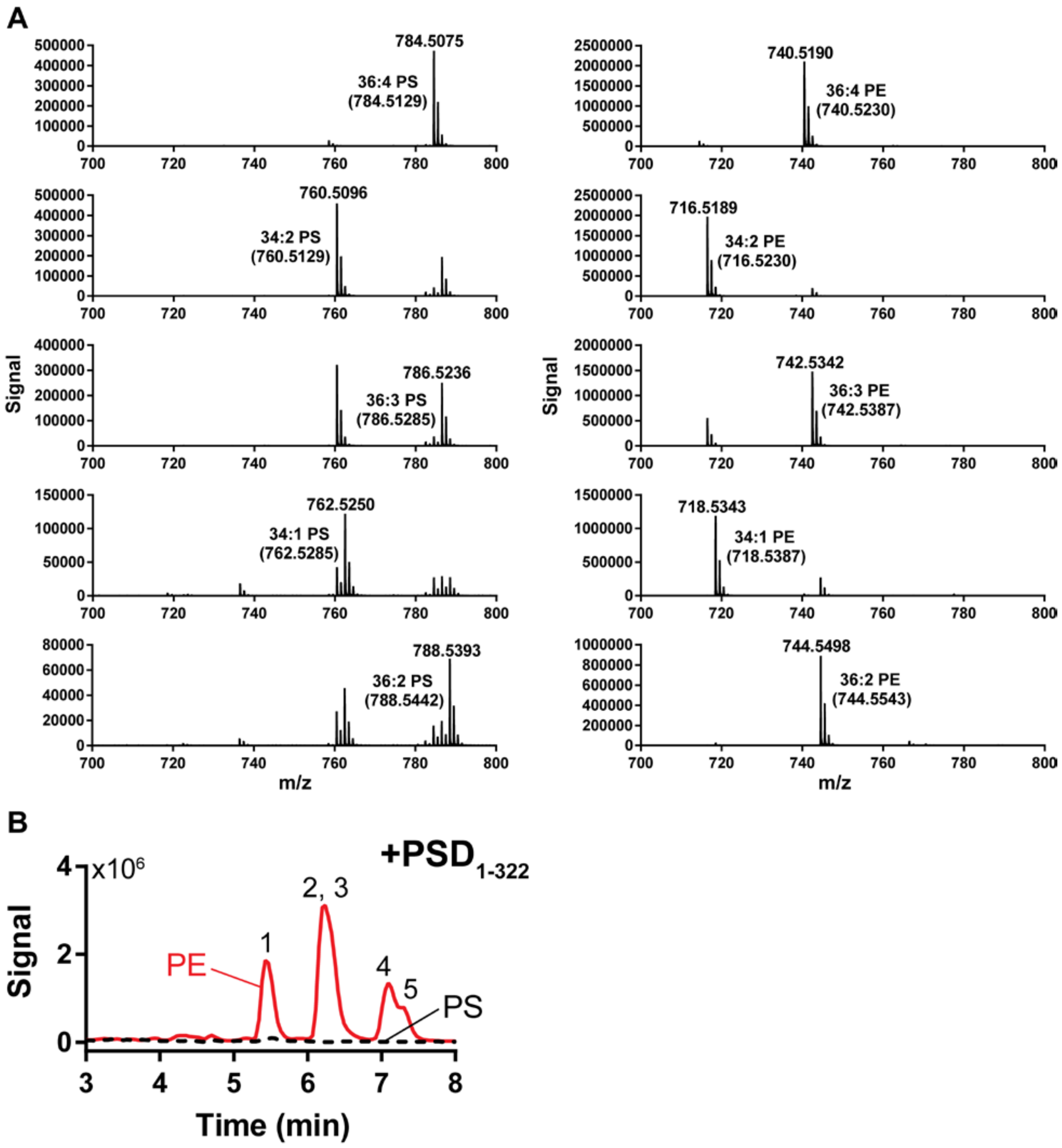

Supplementary Figure S2. LC-MS analysis of the decarboxylation activity of PSD. (A) Identification of phospholipids by LC-MS. MS signals of PS from control reaction of Figure 1B were identified (left panel)**;** signals of PE from PSD$_{1\text{-}287}$ reaction of Figure 1B were identified (right panel). Theoretical m/z values of the molecules are given in parentheses, while observed

values are labeled on top of each peak. **(B)** An analysis of PS decarboxylase activity of $PSD_{1\text{-}322}$ related to figure 1B. Numbers in the chromatogram indicates individual PE molecular species: 1-5 represents 36:4, 34:2, 36:3, 34:1, and 36:2 PE, respectively.

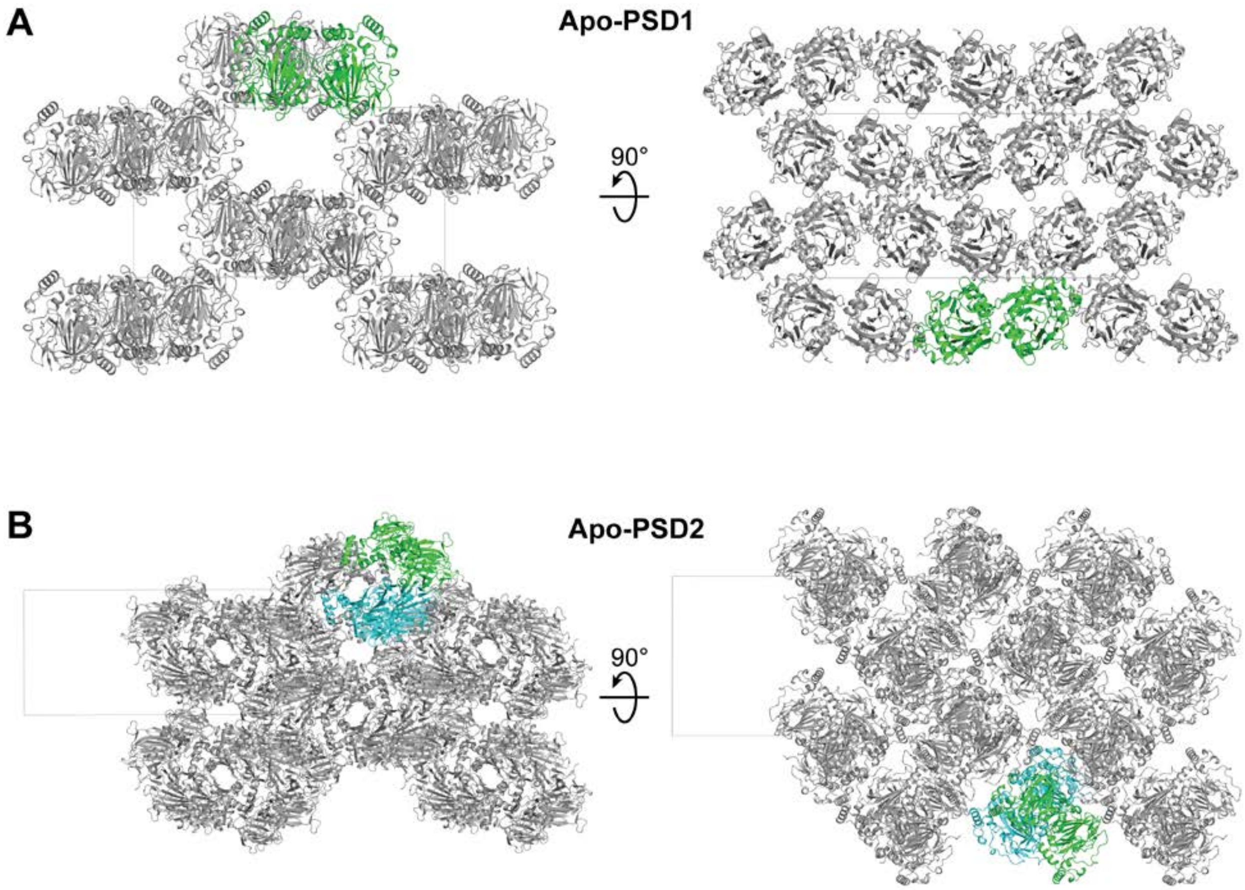

Supplementary Figure S3. Distinct crystal packing of Apo-PSD. Apo-PSD1 **(A)** and Apo-PSD2 **(B)** are shown in two different views. Gray rectangles indicate unit cell. Asymmetric unit of each crystal is highlighted: two protomers in Apo-PSD1 are colored in green; four protomers in Apo-PSD2 are colored in green or cyan.

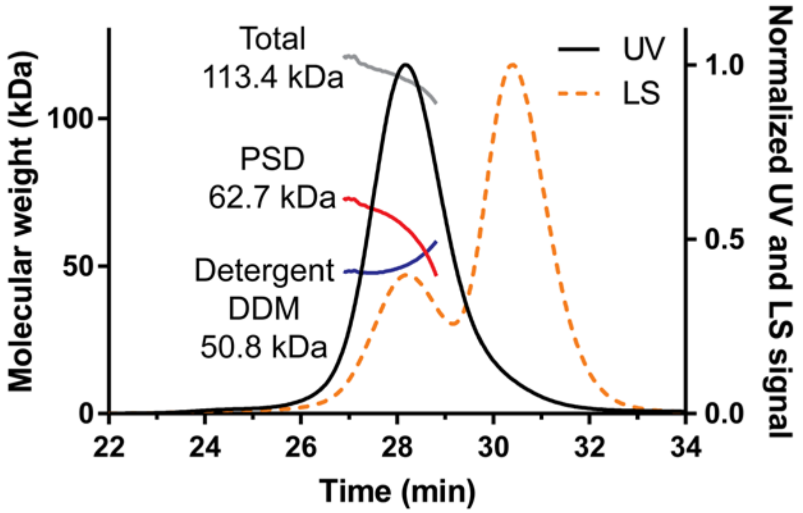

Supplementary Figure S4. PSD$_{1-287}$ forms a dimer in solution. Purified proteins were applied to size exclusion column and eluents were analyzed by multi angle light scattering (MALS) equipped with UV detector. Theoretical molecular weight of the protein is 33.1 kDa.

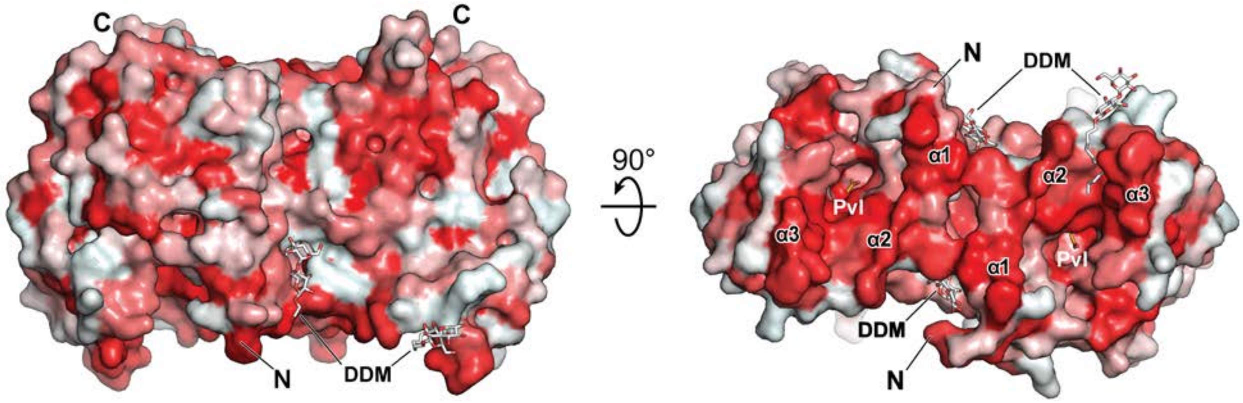

Supplementary Figure S5. N-terminal three-helices are hydrophobic. Hydrophobicity are mapped on the surface of Apo-PSD1 in hydrophobic (red) to non-hydrophobic (white) gradient. The hydrophobicity coloring was generated according to color h of PyMOL tutorial.

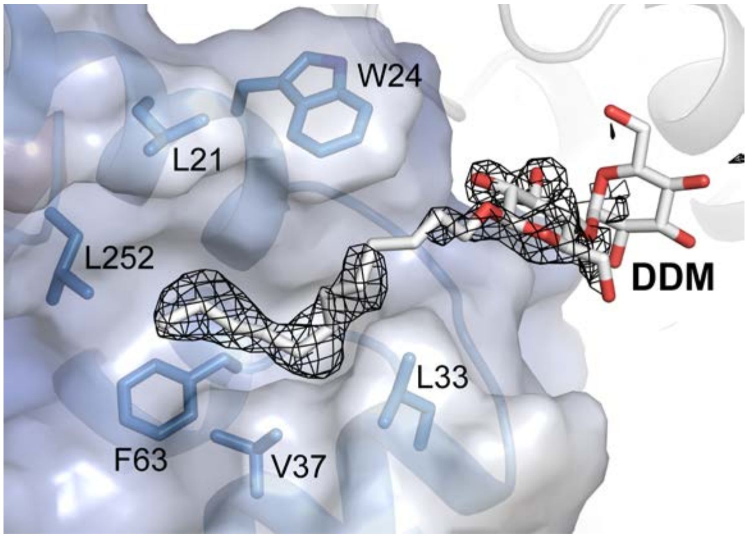

30 **Supplementary Figure S6. Identification of DDM in the hydrophobic pocket.** DDM molecule and the surrounding hydrophobic residues are depicted as sticks. An *Fo-Fc* omit map of DDM is shown in black mesh contoured at 2.5 σ.

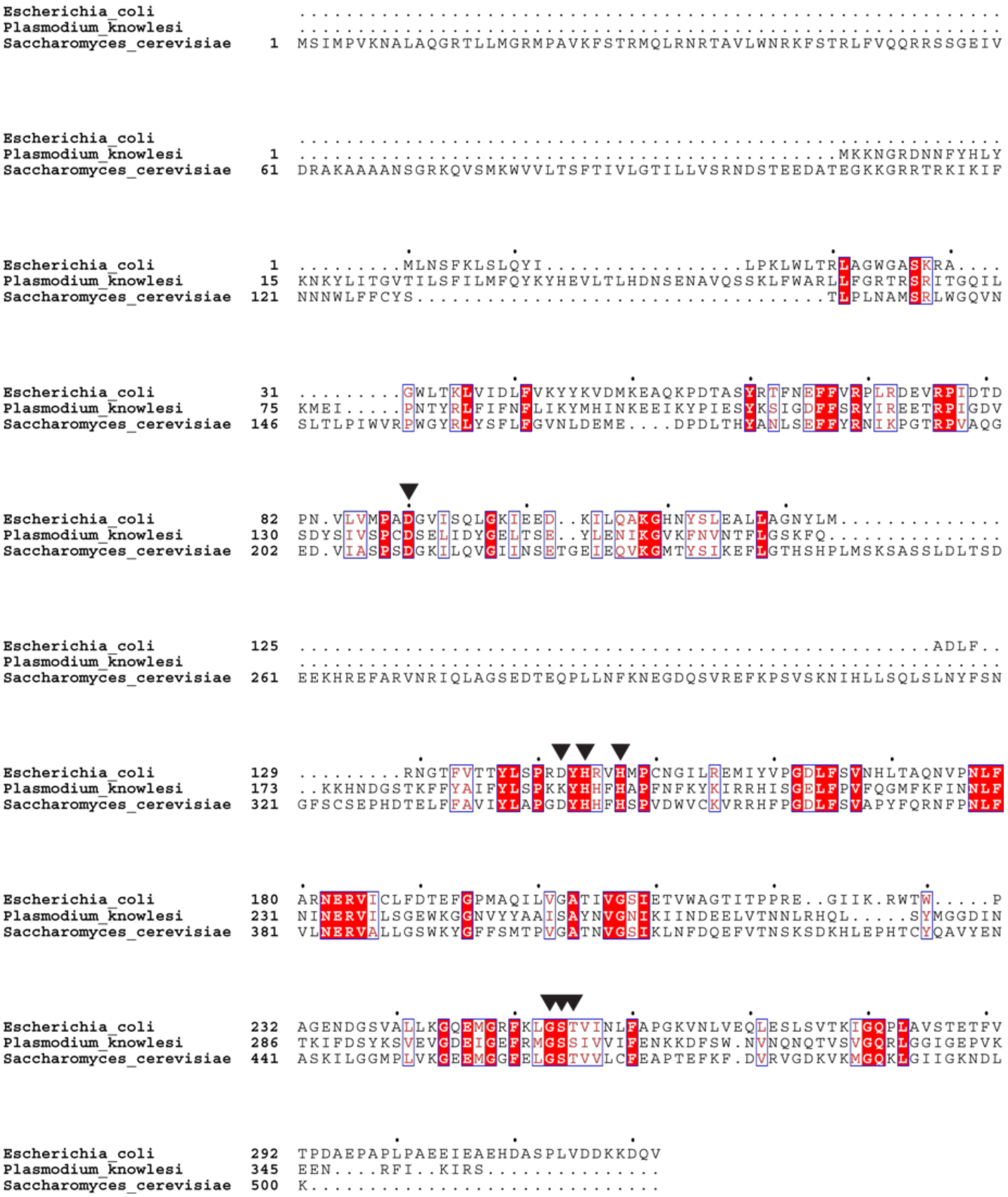

Supplementary Figure S7. Multiple sequence alignment of PSD related to Fig. 6. Sequences of PSD from three homologues (UniProt accession ID: PSD_ECOLI, PSD_PLAKH, and PSD1_YEAST) are aligned and visualized as that of Supplementary Fig. S1. Residues targeted for site-directed mutagenesis of auto-cleavage are designated in arrow.

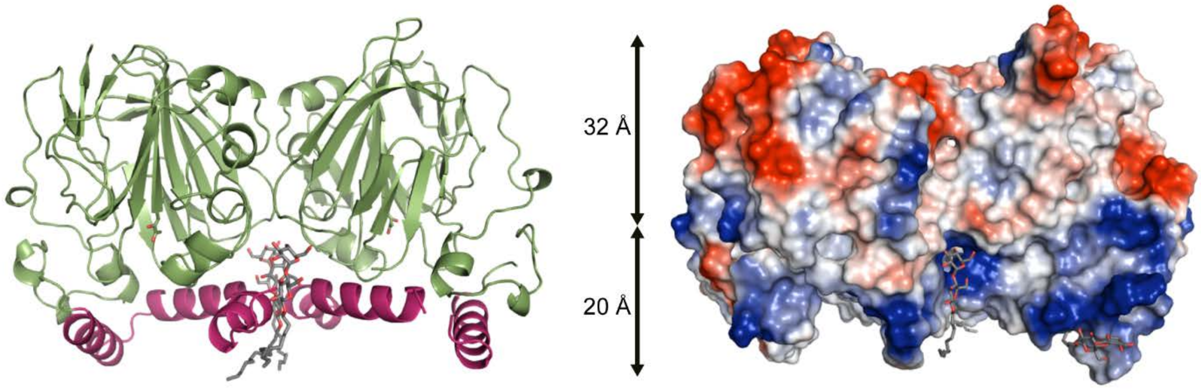

Supplementary Figure S8. Model of membrane-binding domain. A cartoon representation of Apo-PSD1 in green with hydrophobic N-terminal helices highlighted in purple (Left). Surface representation of the enzyme on which electrostatic potential is mapped, where positive charges are colored in blue, negative in red, and hydrophobic surfaces in white (Right). DDM molecules are presented as black stick.

Raw_image of Fig 3B

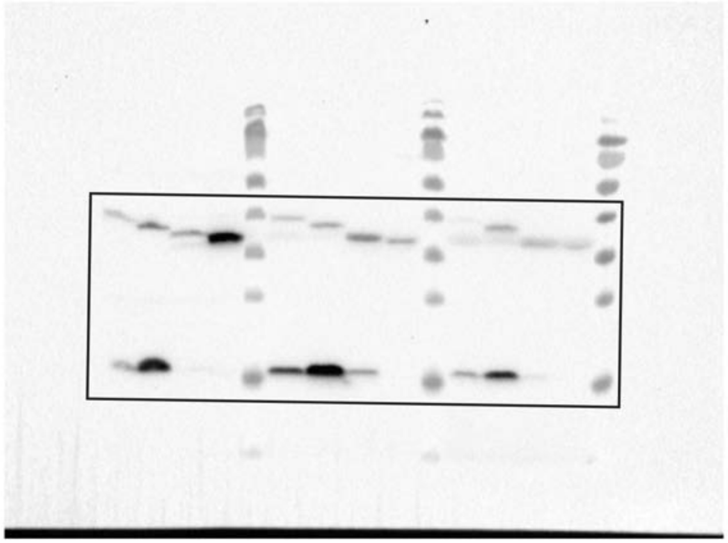

Raw_image of Fig 6A_1

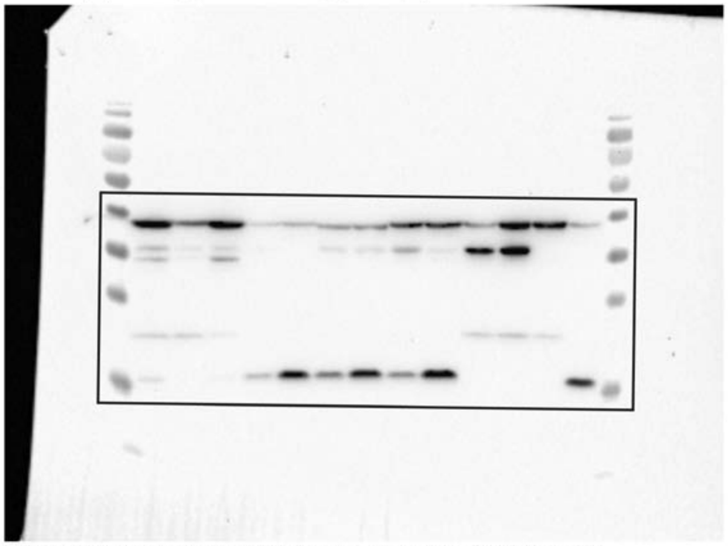

* Illustrator was used to flip the western blot horizontally

(For figure caption see next page)

Raw_image of Fig 6A_2

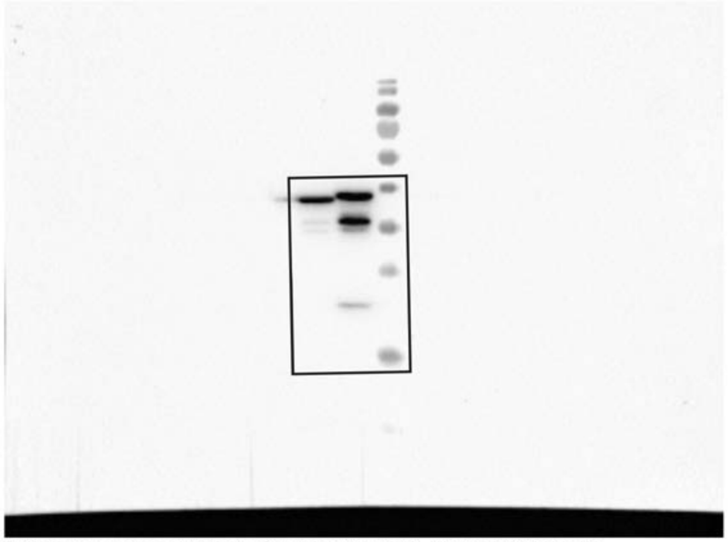

* Illustrator was used to flip the western blot horizontally

Raw_image of Fig 6A_3

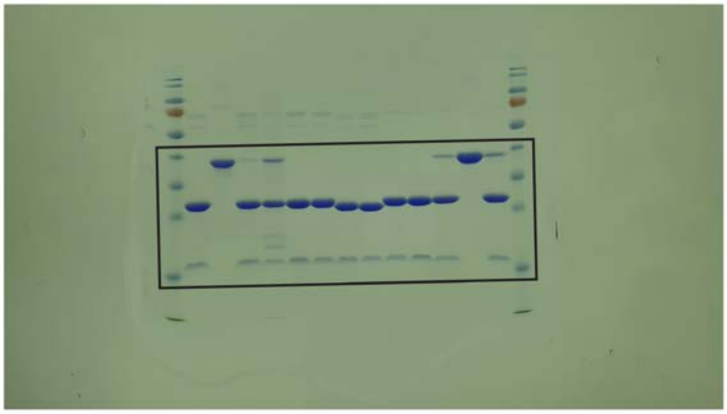

(For figure caption see next page)

Raw_image of Fig 6A_4

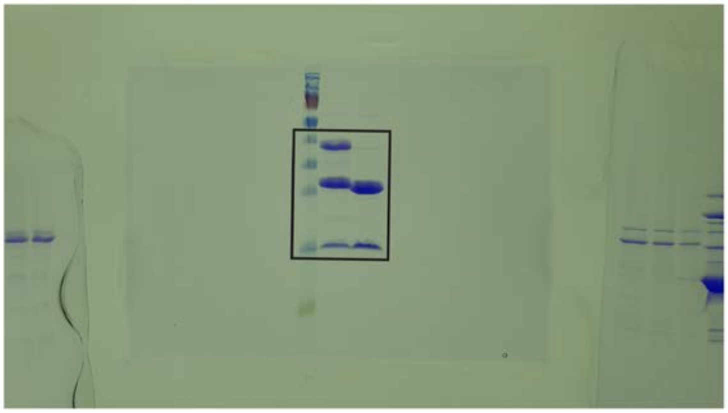

Supplementary Figure S9. Uncropped raw images of the Fig 3B and Fig 6A. First three panels show the original images of Western blots in **Figs. 3B** and **6A**, focusing on the area containing the blots. As a result, certain lengths of the membrane are not shown. The last two show the original images of the SDS-gel in Figure 6A.

Supplementary Table S1. Primers used in this study

No.	Plamsid	Template	Primer name	Primer sequence
1	pLATE31-PSD1-322	JW4121-AM, NBRP, Japan	PSD_ECOLI_1-322_pLATE31	Forward: 5'-AGAAGGAGATATAACT **ATG** TTAAATTCATTTAAACTTTCGCTACAG-3' Reverse: 5'-GTG GTG GTG ATG GTG ATG GCC GACCTGGTCTTTTTTGTCGTCAAC-3'
2	pLATE31-PSD1-287	pLATE31-PSD1-322	PSD_ECOLI_1-287_pLATE31	Forward: same as PSD_ECOLI_1-322_pLATE31_Forward Reverse: 5'-GTG GTG GTG ATG GTG ATG GCC GGTAGATACTGCCAGCGG-3'
3	pLATE31-PSD13-322	pLATE31-PSD1-322	PSD_ECOLI_pLATE31_DelH1	Forward: 5'-AGAAGGAGATATAACT **ATG** CTGCCGAAACTATGGCTTACTC-3' Reverse: same as PSD_ECOLI_1-322_pLATE31_Reverse
4	pLATE31-PSD30-322	pLATE31-PSD1-322	PSD_ECOLI_pLATE31_DelH12	Forward: 5'-AGAAGGAGATATAACT **ATG** GCAGGATGGCTGACAAAACTG-3' Reverse: same as PSD_ECOLI_1-322_pLATE31_Reverse
5	pLATE31-PSD46-322	pLATE31-PSD1-322	PSD_ECOLI_pLATE31_DelH123	Forward: 5'-AGAAGGAGATATAACT **ATG** AAGGTCGACATGAAAGAGGCG-3' Reverse: same as PSD_ECOLI_1-322_pLATE31_Reverse
6	pLATE31-PSD_S166A	pLATE31-PSD1-322	PSD_ECOLI_S166A_Quik_t496g	Forward: 5'-ccgggcgatctcttcGccgttaaccatctca-3' Reverse: 5'-tgagatggttaacggCgaagagatcgcccgg-3'
7	pLATE31-PSD_Y137F	pLATE31-PSD1-322	PSD_ECOLI_Y137F_Quik_a410t	Forward: 5'-cgtttgtgaccacttTcctctccccgcgt-3' Reverse: 5'-acgcggggagaggAaagtggtcacaaacg-3'
8	pLATE31-PSD_Y137F/S166A	pLATE31-PSD_Y137F	PSD_ECOLI_S166A_Quik_t496g	Forward: 5'-ccgggcgatctcttcGccgttaaccatctca-3' Reverse: 5'-tgagatggttaacggCgaagagatcgcccgg-3'
9	pLATE31-PSD_H144A	pLATE31-PSD1-322	PSD_ECOLI_H144A_Quik_c430g_a431c	Forward: 5'-ccccgcgtgactacGCccgcgtacacatgc-3' Reverse: 5'-gcatgtgtacgcggGCgtagtcacgcgggg-3'
10	pLATE31-PSD_H144N	pLATE31-PSD1-322	PSD_ECOLI_H144N_Quik_c430a	Forward: 5'-ccccgcgtgactacAaccgcgtacacatg-3' Reverse: 5'-catgtgtacgcggtTgtagtcacgcgggg-3'
11	pLATE31-PSD_S254A	pLATE31-PSD1-322	PSD_ECOLI_S254A_Quik_t760g	Forward: 5'-aatgggtcgctttaaactcggtgccaccgttatca-3' Reverse: 5'-tgataacggtggcaccgagtttaaagcgacccatt-3'
12	pLATE31-PSD_H147N	pLATE31-PSD1-322	PSD_ECOLI_H147N_Quik_c439a	Forward: 5'-gtgactaccaccgcgtaAacatgccgtgcaac-3' Reverse: 5'-gttgcacggcatgtTtacgcggtggtagtcac-3'
13	pLATE31-PSD_H147A	pLATE31-PSD1-322	PSD_ECOLI_H147A_Quik_c439g_a440c	Forward: 5'-gactaccaccgcgtaGCcatgccgtgcaacgg-3' Reverse: 5'-ccgttgcacggcatgGCtacgcggtggtagtc-3'
14	pLATE31-PSD_D90N	pLATE31-PSD1-322	PSD_ECOLI_D90N_Quik_g268a	Forward: 5'-ctggtcatgcctgccAatggcgttatcagcc-3' Reverse: 5'-ggctgataacgccatTggcaggcatgaccag-3'
15	pLATE31-PSD_D90A	pLATE31-PSD1-322	PSD_ECOLI_D90A_Quik_a269c	Forward: 5'-ggtcatgcctgccgCtggcgttatcagcc-3' Reverse: 5'-ggctgataacgccaGcggcaggcatgacc-3'
16	pLATE31-PSD_D142N	pLATE31-PSD1-322	PSD_ECOLI_D142N_Quik_g424a	Forward: 5'-acctctccccgcgtAactaccaccgc-3' Reverse: 5'-gcggtggtagtTacgcggggagaggt-3'
17	pLATE31-PSD_D142A	pLATE31-PSD1-322	PSD_ECOLI_D142A_Quik_a425c	Forward: 5'-ctccccgcgtgCctaccaccgcg-3' Reverse: 5'-cgcggtggtagGcacgcggggag-3'
18	pLATE31-PSD_T255A	pLATE31-PSD1-322	PSD_ECOLI_T255A_Quik_a763g	Forward: 5'-cgctttaaactcggttccGccgttatcaacctgtttg-3' Reverse: 5'-caaacaggttgataacggCggaaccgagtttaaagcg-3'
19	pLATE31-PSD_H144A/H147A	pLATE31-PSD_H144A	PSD_ECOLI_H144A/H147A_Quik_c439g_a440c	Forward: 5'-actacGCccgcgtaGCcatgccgtgcaacg-3' Reverse: 5'-cgttgcacggcatgGCtacgcggGCgtagt-3'
20	pLATE31-PSD_D90A/D142A	pLATE31-PSD_D90A	PSD_ECOLI_D142A_Quik_a425c	Forward: 5'-ctccccgcgtgCctaccaccgcg-3' Reverse: 5'-cgcggtggtagGcacgcggggag-3'
21	pLATE31-PSD_G253P	pLATE31-PSD1-322	PSD_ECOLI_G253P_Quik_g757c_g758c	Forward: 5'-aatgggtcgctttaaactcCCttccaccgttatcaacctg-3' Reverse: 5'-caggttgataacggtggaaGGgagtttaaagcgacccatt-3'
22	pLATE31-PSD_G253A	pLATE31-PSD1-322	PSD_ECOLI_G253A_Quik_g758c	Forward: 5'-gggtcgctttaaactcgCttccaccgttatcaacc-3' Reverse: 5'-ggttgataacggtggaaGcgagtttaaagcgaccc-3'

References

1. Madeira, F. *et al.* The EMBL-EBI search and sequence analysis tools APIs in 2019. *Nucleic Acids Res.* (2019) doi:10.1093/nar/gkz268.

2. Robert, X. & Gouet, P. Deciphering key features in protein structures with the new ENDscript server. *Nucleic Acids Res.* (2014) doi:10.1093/nar/gku316.
